# Synthesis of a guanylylated peptide derived from norovirus VPg as a molecular tool for studying norovirus replication

**DOI:** 10.1039/d6cb00092d

**Published:** 2026-08-10

**Authors:** Bob van Puffelen, Leandro Xavier Neves, Roy Steneker, Nico J. Meeuwenoord, Edward Emmott, Dmitri V. Filippov

**Affiliations:** a Leiden Institute of Chemistry, Leiden University 2333 CC Leiden The Netherlands; b Centre for Proteome Research, Faculty of Health and Life Sciences, University of Liverpool Liverpool L69 7ZB UK e.emmott@liverpool.ac.uk; c Department of Biochemistry, Cell & Systems Biology, Institute of Systems, Molecular & Integrative Biology, Faculty of Health and Life Sciences, Biosciences Building, Crown Street, University of Liverpool Liverpool L69 7ZB UK

## Abstract

Norovirus is a leading cause of global acute gastroenteritis, yet the mechanisms underlying its replication remain incompletely understood. As a positive-sense single-stranded RNA (+ ssRNA) virus, norovirus utilizes a nucleotidylated viral protein (VPg) as a primer for synthesis of its genomic and subgenomic mRNAs. In this study, we report the efficient solid-phase synthesis of a guanylylated (also known as GMPylated) VPg-derived peptide fragment using a novel pre-guanylylated tyrosine building block equipped with acid-sensitive protecting groups. This approach enables a streamlined, one-step deprotection and cleavage process. The resulting synthetic peptide was characterised *via* LC-MS/MS, establishing its gas-phase fragmentation behaviour and confirming prior observations of a diagnostic guanine nucleobase peak at 152.0572 *m*/*z*. We subsequently applied these findings to develop a parallel reaction monitoring (PRM) assay to investigate endogenous VPg guanylylation in murine microglial cells infected with murine norovirus (MNV). Our results demonstrate that while unmodified VPg is detectable from 4 hours post-infection, the guanylylated form appears predominantly during later stages (8–12 hpi). The timing of VPg guanylylation is consistent with a model where guanylylation may serve as a regulator in the switch between anti-sense and sense RNA synthesis, though at this time this hypothesis is speculative and remains to be proven. These synthetic molecular tools and mass spectrometry assays provide a robust framework for further exploring the role of nucleotidylation in viral pathogenesis and in broader cell and pathogen biology.

## Abstract 2

Norovirus is a leading cause of global acute gastroenteritis, yet the mechanisms underlying its replication remain incompletely understood.

## Graphical abstract

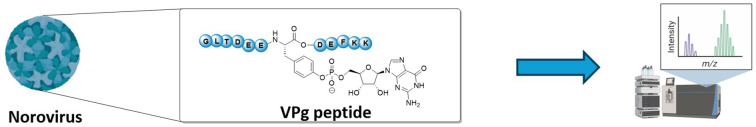

## Introduction

Norovirus, a member of the *Caliciviridae* family, is a positive-sense single-stranded RNA (+ ssRNA) virus and a leading cause of acute gastroenteritis worldwide (winter vomiting disease), especially affecting young children and the elderly.^1^ Due to a lack of specific treatment, norovirus infection causes 200 000 deaths each year and imposes an economic burden exceeding $60 billion.^2–4^ To address this challenge and develop medicines and diagnostics, further research into norovirus reproduction is needed.^5^

**Fig. 1:**
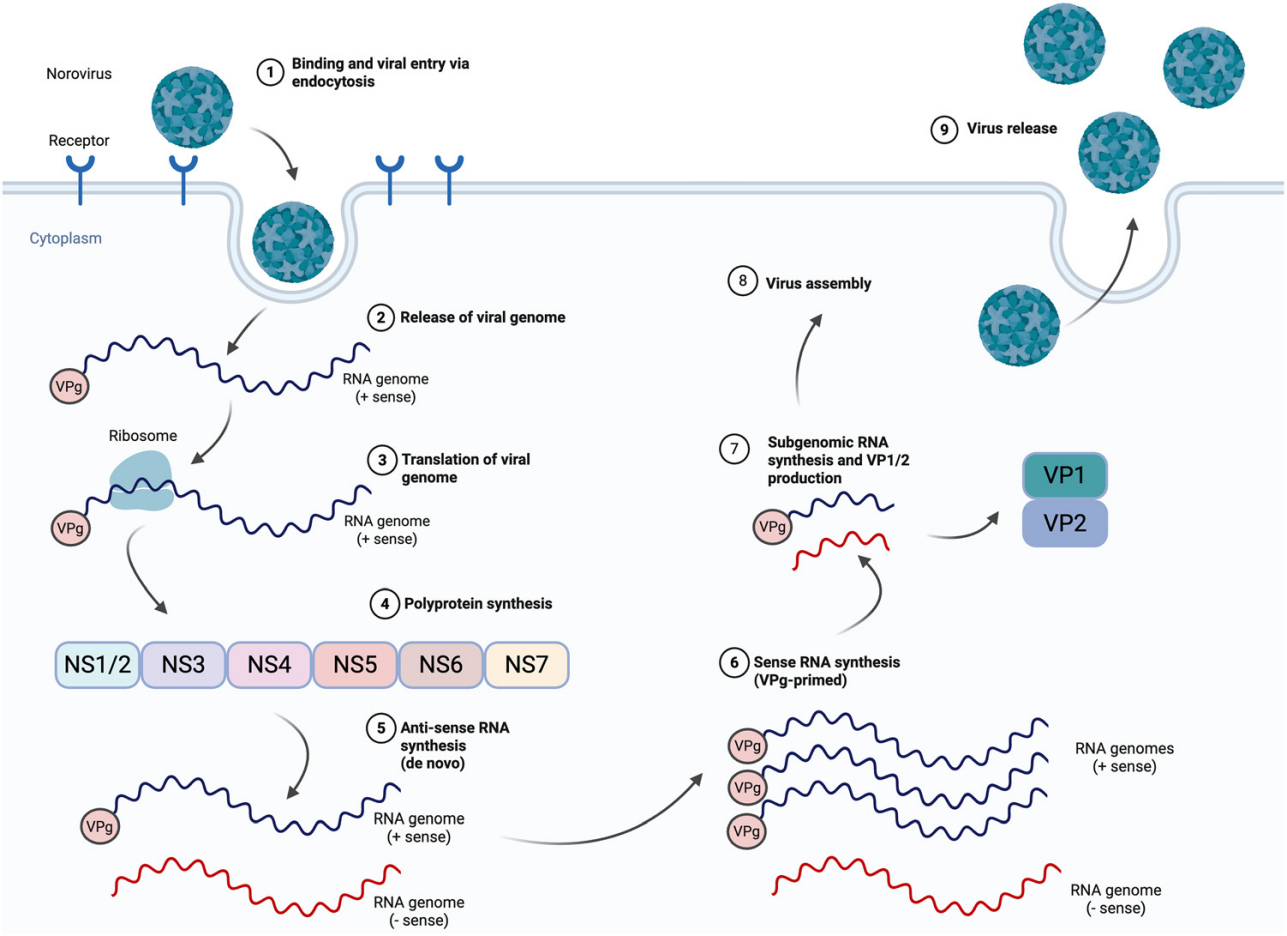
The norovirus replication cycle. (1) Norovirus first binds and enters the cell through receptor-mediated endocytosis. (2) The viral genome is released into the cytoplasm, where (3) it is translated in a VPg-dependent manner to produce (4) the viral polyprotein. The viral polyprotein is post-translationally cleaved by the viral NS6 protein to produce the various functional components required for replication, including VPg (NS5). (5) Anti-sense RNA is produced, in a *de novo*-primed manner. This is used as a template for (6) VPg-primed synthesis of positive-sense viral RNAs. (7) The virus also produces a shorter subgenomic mRNA, from which the major (VP1) and minor (VP2) capsid proteins are produced. (8) Genomic mRNAs are packaged into virions and (9) released from the cell. This figure was produced in Biorender.

The replication cycle of norovirus (Fig. 1) offers key targets for intervention as a potential therapeutic strategy, including the viral protease and polymerase proteins and their activities. The virus must first enter the cell, *via* receptor-dependent endocytosis, which in the case of murine norovirus (MNV) employs murine CD300LF.^6,7^ After entry, the viral genome is released. The norovirus genome is polyadenylated but covalently linked to a viral protein (VPg: Viral Protein genome-linked) at its 5′ end, with VPg recruiting the host translational machinery to permit initial translation of the viral polyprotein.^8,9^ The polyprotein consists of 6 domains: NS1/2, NS3, NS4, NS5 (VPg), NS6 (Pro) and NS7 (Pol). These are cleaved by the viral protease NS6 in a temporally regulated manner to yield the proteins needed for replication of the viral genome.^10,11^ Initially, anti-sense RNA is produced as a replication intermediate, with current evidence supporting *de novo* rather than VPg-primer production of anti-sense RNAs.^12,13^ Later in infection, the virus switches to production of sense RNAs.^14^ As depicted in Fig. 1, norovirus uses a nucleotidylated VPg, to initiate replication of its sense RNAs: the genome and subgenomic mRNA. Nucleotidylation is catalysed by the viral polymerase or its pro-pol precursor.^15,16^ The nucleotidylated forms of VPg serve as primers for sense RNA replication.^12,17^ Another well-known example of a virus that uses a similar mechanism is poliovirus, where the uridylylated VPg, or rather its dinucleotidylated form (VPgpUpU), functions as the replication primer.^18^ The genomic RNA of norovirus and poliovirus is linked by a phosphodiester bond between the 5′-terminal guanosine (norovirus) or uridine (poliovirus) and the phenolic oxygen of a tyrosine residue (Y27 for human norovirus and Y26 for murine norovirus).^15,16^ When involving guanosine, this post-translational modification (PTM) is variously termed guanylylation or GMPylation. The ability to produce synthetic nucleotidylated VPg, or peptides derived from it can advance our understanding of these key stages of norovirus replication. Production of the subgenomic mRNA leads to expression of the VP1 and VP2 capsid proteins, and in the case of murine norovirus the VF-1 protein.^19^ Expression of the capsid proteins permits viral assembly and release from cells, concluding the replication cycle.

In the past, we synthesized VPgs from poliovirus and other nucleotidylated peptides on solid phase using pre-nucleotidylated building blocks.^20–23^ Here we present the synthesis of a peptide derived from guanylylated norovirus VPg (1 in Scheme 2), characterisation of its gas-phase behaviour and fragmentation properties. We then apply this synthetic guanylylated VPg-peptide to support investigation of guanylylation in the context of authentic murine norovirus replication.

## Results and discussion

**Scheme 1:**
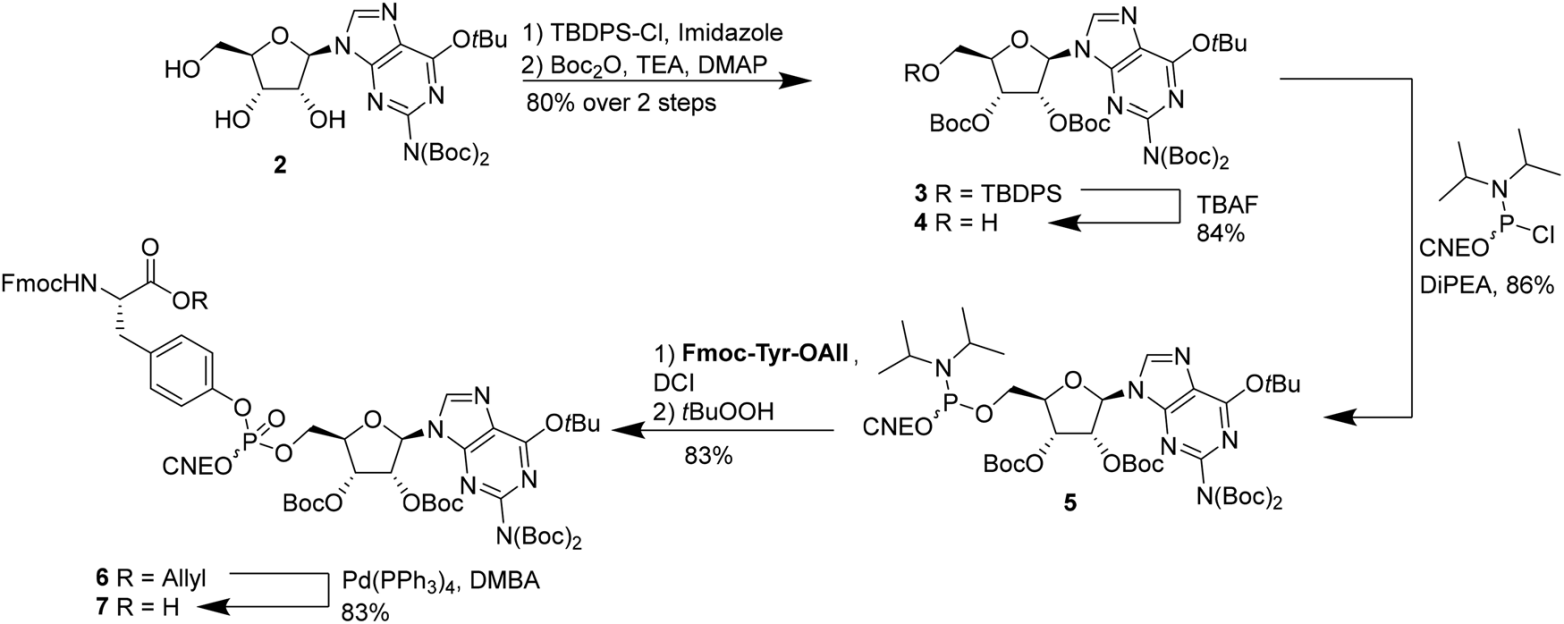
The synthesis of guanylylated tyrosine residue 7 for solid-phase peptide synthesis.

**Scheme 2:**
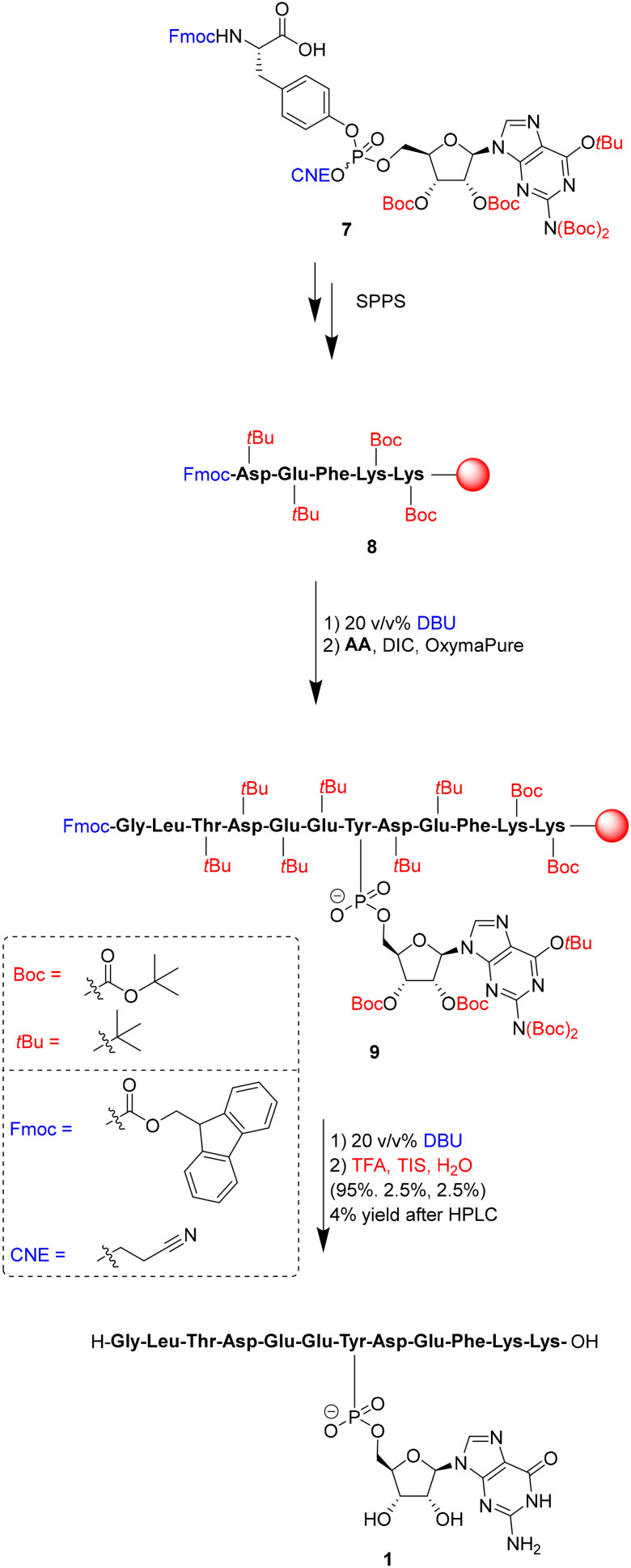
The solid-phase synthesis of VPg peptide fragment 1.

Based on our previous experience with poliovirus VPg, we decided to synthesize noroviral VPg fragment 1 (Scheme 2) suitable as a reference compound for bioanalytical studies using a pre-nucleotidylated Fmoc-tyrosine building block 7 (Scheme 1).^20–23^ Unlike in the previously reported procedures, we used a building block equipped with acid-sensitive protecting groups (Boc/*t*-Bu) to enable a one-step deprotection process that simultaneously removes the side-chain protecting groups and cleaves the product from the resin. The exocyclic amine and oxygen functionalities of the guanine base were protected with *tert*-butyloxycarbonyl (Boc) and *tert*-butyl groups, respectively, to generate guanosine derivative 2 according to a previously reported procedure.^24^ This allowed for further protecting group manipulations, beginning with regioselective silylation of the 5′-OH, followed by the protection of the remaining hydroxyl groups with Boc, affording guanosine 3 in 80% yield over two steps. Subsequent removal of the 5′-silyl group with TBAF liberated the 5′-OH, enabling installation of the phosphoramidite moiety in 86% yield. Phosphoramidite 5 was then coupled to Fmoc-Tyr-OAll using 4,5-dicyanoimidazole (DCI) as the activator, and the resulting phosphite triester was subsequently oxidized to its corresponding phosphotriester 6 in 83% yield. Finally, the allyl ester was removed using a catalytic amount of Pd(PPh_3_)_4_ and dimethyl barbituric acid (DMBA) to yield carboxylic acid 7 in 83% yield, making it suitable for solid-phase synthesis.

With carboxylic acid 7 in hand, we turned our attention to the synthesis of protected and immobilized pentapeptide 8 (Scheme 2). To achieve this, we employed a TentaGel S PHB resin pre-loaded with lysine. Automated Fmoc-based solid-phase peptide synthesis (SPPS) at elevated temperature with commercially available, aptly protected amino acids and DIC/OxymaPure as the coupling reagent yielded immobilized peptide 8. Guanylylated tyrosine 7 was introduced using the same coupling protocol at room temperature, after which the Fmoc and 2-cyanoethyl (CNE) groups were removed with 2% DBU in DMF. The peptide was further elongated in a similar fashion to provide the protected guanylylated peptide 9. Treatment of 9 with DBU and TFA, sequentially, cleaved the final Fmoc group, freed the immobilized peptide from the resin, and unmasked the protected functional groups, resulting in the target peptide 1 in an overall yield of 4% after HPLC purification. This relatively low yield may be contributed to losses during HPLC recovery, as the LC-MS trace of the crude peptide is of good quality (Fig. S5).

**Fig. 2:**
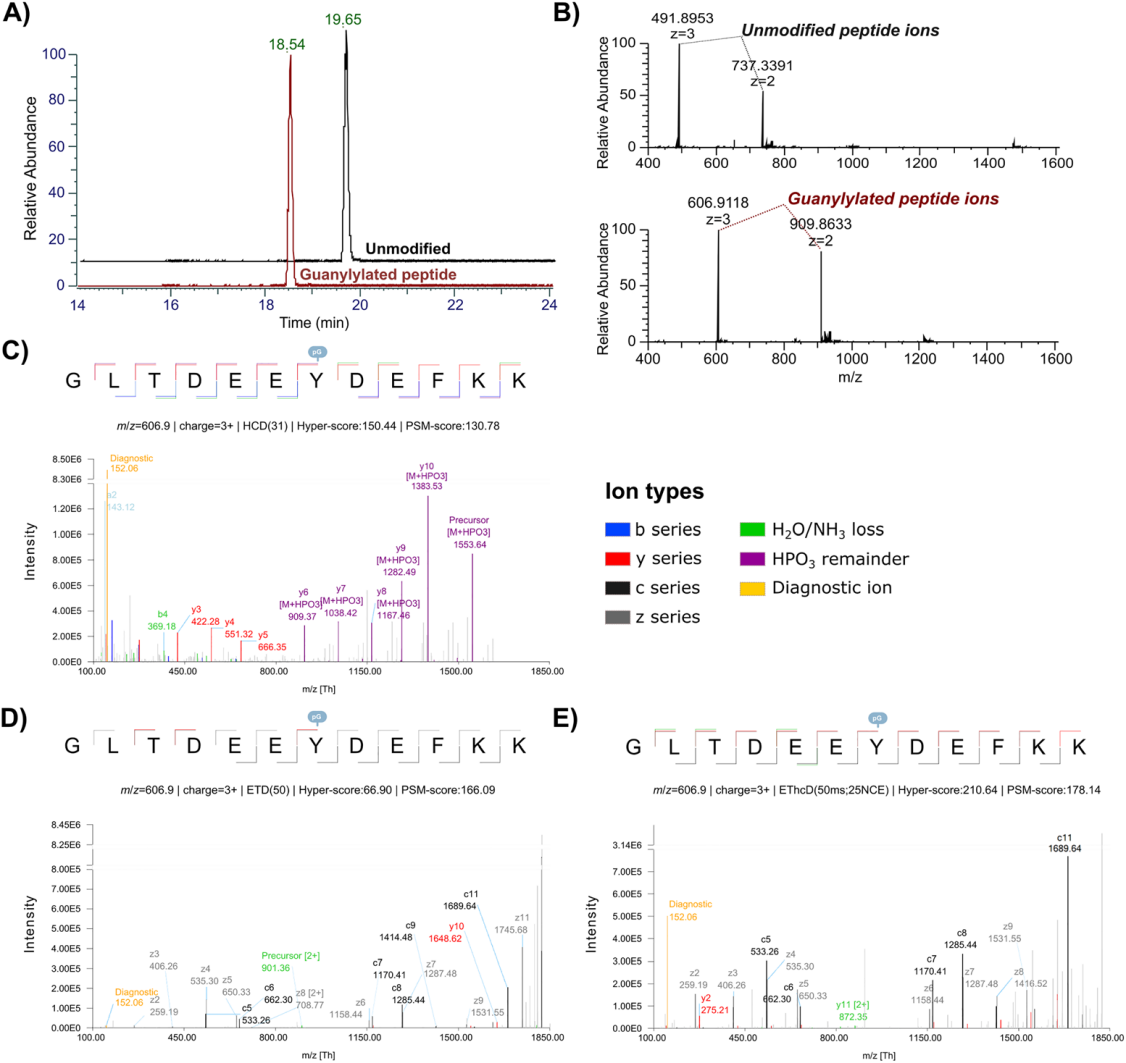
LC-MS/MS characterisation of guanyl-GLTDEEYDEFKK synthetic peptide. (A) C18 reversed-phase chromatography indicating reduced hydrophobicity of guanylylated peptide (red) compared to the unmodified sequence (black). (B) Peptide ions observed as triply and doubly charged species confirm a 345.047 Da mass delta in the modified peptide. (C)–(E) Representative MS/MS spectra of the guanlylyl-modified peptide under optimised HCD (C), ETD (D) and EThcD (E) activation.

The synthetic VPg-peptide was evaluated by LC-MS to characterise elution profiles during C18 reversed-phase chromatography and by tandem mass spectrometry. The guanylylated VPg peptide exhibited slightly reduced hydrophobicity when compared to an unmodified peptide (Fig. 2A), and following electrospray ionisation (ESI), both unmodified and modified peptides were observed as triply and doubly charged ion species in the gas phase. High-resolution/accurate mass (HRAM) mass spectrometry analysis confirmed a mass delta of 345.047 Da (H_12_C_10_PO_7_N_5_) in the modified peptide compared to its unmodified counterpart, matching the guanylyl modification introduced to the tyrosine (Fig. 2B). Furthermore, gas-phase mobility characteristics were evaluated by FAIMS (Field asymmetric waveform ion mobility spectrometry). The optimal compensation voltages (CVs) for the guanylylated peptide were 3 V and 8 V lower than those of the 2+ and 3+ unmodified peptide, respectively (Fig. S1).

MS/MS spectra were acquired for the modified peptide under HCD (higher-energy collisional dissociation), ETD (electron-transfer dissociation), and EThcD (combined ETD and HCD) activation methods. Under optimised normalised collisional energy (NCE) and reaction times, high-quality MS/MS spectra could be obtained for all activation methods (Fig. 2C–E). Nearly full sequence coverage of the peptide sequence was achieved through *b* and *y* ion series with HCD activation (NCE = 31), or *c* and *z* ion series with ETD (50 ms), or all four (*b*, *c*, *y*, *z*) ion series with EThcD (50 ms, NCE = 25). Notably, all dissociation methods produced site-specific fragment ions enabling the guanylyl modification to be confidently localised in the tyrosine residue. Also, we observed a neutral loss of 265.0811 Da (H_11_O_4_C_10_N_5_) under HCD activation indicating a partial breakdown of GMP but leaving a HPO_3_-remainder moiety attached to the tyrosine residue (Fig. 2C, purple peaks). In line with observations from coronavirus guanylylation, the dominant feature of the HCD spectra, and evident, but to a much lesser extent in the EThcD spectra, was a strong diagnostic peak at 152.0572 *m*/*z* resulting from fragmentation of the GMP, yielding the guanine nucleobase (C_5_H_5_N_5_O).^25^

**Fig. 3:**
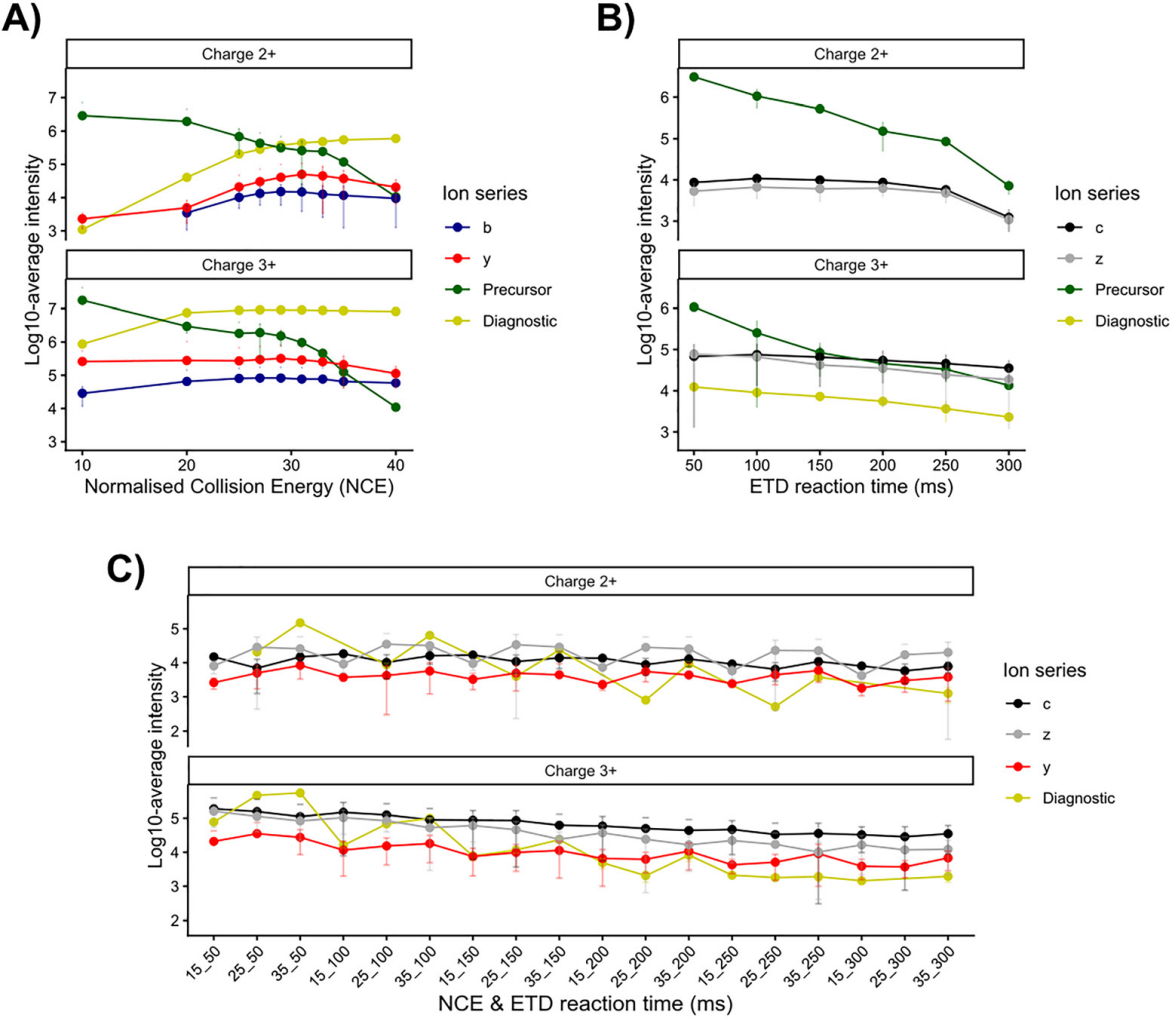
Efficiency of HCD, ETD, and EThcD settings for MS/MS analysis of guanylylated peptide. (A) NCE values around 30% yielded MS/MS spectra with intense *b*/*y* ion series and diagnostic ions. (B) ETD reaction times showed a broader effective range, with the highest average *c*/*z* fragment intensities observed between 50–100 ms, followed by a gradual decline at longer times. Overall, fragment intensities were an order of magnitude higher when analyzing the triply charged precursor (606.9118 *m*/*z*, 3+). (C) EThcD fragmentation produced the highest average *c*/*z*/*y* fragment intensities at short ETD reaction times (50–100 ms) when combined with an HCD NCE of 25%. As with ETD, fragment intensities were an order of magnitude higher for the triply charged precursor (606.9118 *m*/*z*, 3+).

Next, we performed a more comprehensive evaluation of optimal NCE and ETD reaction times when fragmenting the guanylylated peptide *via* HCD- and ETD-based methods, respectively, or a combination of both *via* EThcD. For HCD only, NCE around 30% achieved the highest average intensity of *b* and *z* ions, with diagnostic ion reaching near-maximum intensity and significant reduction of non-fragmentated peptide precursor (Fig. 3A). Overall, the triply charged peptide precursor yielded a higher average intensity of fragment ions compared to the doubly charged precursor, although PSM scores were similarly high in both cases (Fig. S2A, SI). ETD reaction times within 50–100 ms yielded slightly more intense fragments, with a gradual decline with higher values (Fig. 3B). Notably, fragment ions from the triply charged precursor were on average 1 order of magnitude higher compared to fragments from the doubly charged precursor. This disparity can be attributed to non-dissociative electron transfer (charge reduction) that knowingly limits backbone cleavage in lower charge states upon ETD activation.^26^ Similarly, in EThcD fragmentation, the triply charged precursor produced higher intensity fragments, and overall fragment intensities were slightly higher within 50–100 ms reaction times, and best when combined with NCE of 25% for 3+ precursor or 35% NCE for 2+ precursor (Fig. 3C). Regardless of the activation method, triply charged precursors exhibited higher PSM scores compared to doubly charged precursors (Fig. S2).

With the synthetic peptides fully characterised by LC-MS/MS, we were able to use these data to develop a parallel reaction monitoring assay (PRM) for monitoring both unmodified and guanylylated forms of VPg during murine norovirus infection. Matrix-matched calibration curves ranging from approximately 25 fmol to 50 amol of synthetic peptides on-column per 300 ng of digest were used to assess the robustness of the assay. Synthetic VPg peptides showed a linear MS response in the range tested (*R*-squared 0.97–0.99; Fig. S3). The comparison of unmodified and guanylylated peptides at the most challenging concentration of the calibration curve (50 amoles) didn't show improvement in the assay sensitivity when FAIMS was used with optimised CV (Fig. S4). For this reason, subsequent PRM assays of MNV time course samples were not performed with FAIMS.

**Fig. 4:**
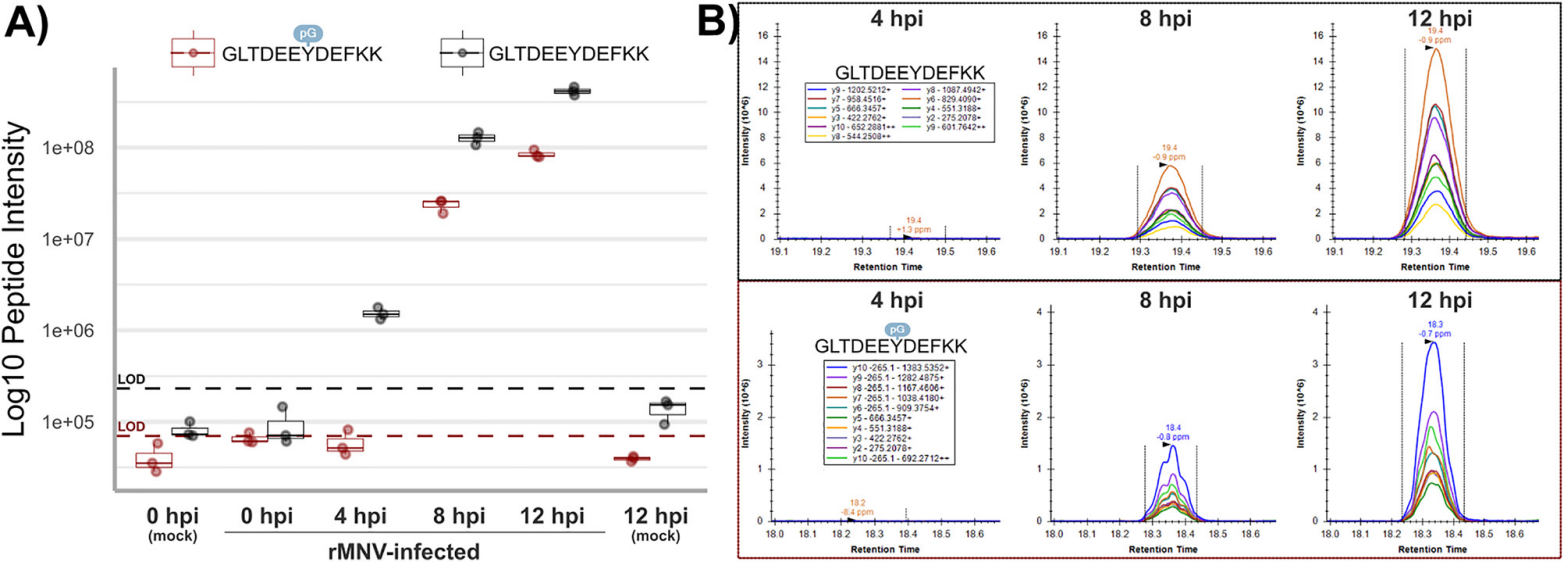
Levels of unmodified and guanylylated VPg peptide during rMNV replication. (A) Unmodified VPg (black) was detected in increasing levels after 4 hpi whereas the endogenous guanylylated VPg (red) was detected at 8–12 hpi only. Dashed lines represent the limit of detection (LOD) of each peptide, defined as the mean of mock-infected samples, plus three standard deviations. (B) Extracted ion chromatograms of unmodified (top) and guanlylyated VPg peptide (bottom) showing the difference in abundance in rMNV-infected samples (4–12 hpi). MNV infections were performed as three independent replicates (*n* = 3).

PRM was applied to investigate the presence of the endogenous guanylylated VPg peptide in BV2 mouse microglial cells lines infected with rMNV. Over the course of 12 hours of infection, VPg could be detected *via* the unmodified GLTDEEYDEFKK from 4 hours post-infection (hpi) onwards, whereas the guanylylated counterpart was only detected in later infection timepoints 8–12 hpi (Fig. 4A and B). The quantification of both unmodified and modified peptides 8–12 hpi enabled us to evaluate relative changes in VPg's occupancy by GMP over time, although no significant differences were observed between those two time points (*t*-test *p*-value = 0.1259). Despite detection of the unmodified VPg peptide at 4 hpi, the guanylylated counterpart was below the limit of detection in two of the three independent infection experiments. Consequently, changes in occupancy between the early (4 hpi) and later (8–12 hpi) stages of the replication cycle could not be reliably assessed. Nonetheless, these preliminary data, although derived from a single data point, suggest that occupancy may be ∼4-fold lower early in infection, warranting confirmatory studies. It should be stressed that absolute occupancy could not be inferred in our analysis due to the absence of standardisation to a stable isotope labelled standard peptide of known abundance. Therefore, our label-free PRM assay is only to estimate changes in the proportion of free and genome-linked VPg across samples without determining stoichiometric ratios of free- to genome-linked VPg. This observed timing of VPg guanylylation correlates with prior observations of temporal regulation of norovirus sense and anti-sense RNA synthesis.^14^ It is tempting to speculate that the availability of nucleotidylated VPg as a primer could serve as a regulator in the sense-antisense switch. Further work to study the regulation and mechanisms of VPg nucleotidylation is needed, and the availability of synthetic nucleotidylated VPg peptides will greatly aid this characterisation, and indeed, the wider role of guanylylation or GMPylation in cell and pathogen biology.

## Conclusion

In conclusion, a guanylylated Fmoc-tyrosine building block with acid-sensitive protection groups was prepared and incorporated into a practical solid-phase procedure to synthesize a VPg fragment of norovirus. The availability of synthetic guanylylated VPg permitted facile characterisation of its properties, with guanylylation inducing shifts in mass-to-charge ratios, retention time and FAIMS CV relative to the unmodified peptide. Further work is required to determine how reproducible these shifts are with different guanylylated peptides. In line with prior reports, the modification was labile under HCD conditions. A full ion series could be observed under HCD conditions, but bearing a 265.0811 neutral loss, leaving a HPO_3_ remnant. An advantage of HCD fragmentation is the production of a dominant diagnostic peak at 152.0572 *m*/*z*, previously observed in studies of coronavirus guanylylation and representing the guanine nucleobase.^25^ In turn, ETD and EThcD permitted observation of the intact modification. Using this knowledge, we established a proteomics-based assay to characterise guanylylated VPg levels during murine norovirus infection. By applying this assay to an infection time course, we found that guanylylated VPg levels peak at mid- to late times post-infection. Such temporal dependence tentatively suggests a role for VPg guanylylation in switching between antisense and sense RNA synthesis.

## Author contributions

Conceptualization, supervision and writing: DVF and EE. Methodology, investigation, data curation and writing: BvP, LXN, RS and NJM.

## Conflicts of interest

The authors have no conflicts of interest.

## Supplementary Material

CB-OLF-D6CB00092D-s001

## Data Availability

The detailed experimental procedures are available in the supporting information (SI), including the spectroscopic characterisation of all synthetic compounds. The mass spectrometry proteomics data have been deposited to the ProteomeXchange Consortium *via* the PRIDE partner repository^27^ with the dataset identifier PXD075074 (PRM) and PXD075200 (synthetic guanylyl peptide). PRM data was also deposited to Panorama Public https://doi.org/10.6069/4zpa-pw40. Annotated MS/MS are openly available in figshare at https://doi.org/10.6084/m9.figshare.31492393. R scripts associated with this manuscript are available through https://github.com/emmottlab/guanylylation_2026. See DOI: https://doi.org/10.1039/d6cb00092d.
