# Supplementary material for: Synthesis of a guanylylated peptide derived from norovirus VPg as a molecular tool for studying norovirus replication
Source: RSC Chem Biol. 2026 Aug 10;7(10):1978–85. doi: 10.1039/d6cb00092d (PMC13485141; doi:10.1039/d6cb00092d)
Supplement: Supplementary file 1 [file CB-007-D6CB00092D-s001.pdf]

Supplementary Information (SI) for RSC Chemical Biology.
This journal is © The Royal Society of Chemistry 2026

Synthesis of a guanylylated peptide derived from norovirus VPg as a molecular tool for studying norovirus replication

Bob van Puffelen[#1], Leandro Xavier Neves[#2,3], Roy Steneker[1], Nico J. Meeuwenoord[1], Edward Emmott*[2,3] & Dmitri V. Filippov*[1]

[#] These authors contributed equally to this work

[1]Leiden Institute of Chemistry, Leiden University, 2333 CC Leiden, The Netherlands

[2]Centre for Proteome Research, Department of Biochemistry, Cell & Systems Biology, Institute of Systems, Molecular & Integrative Biology, University of Liverpool, Liverpool, UK

[3]Department of Biochemistry, Cell & Systems Biology, Institute of Systems, Molecular & Integrative Biology, Faculty of Health and Life Sciences, Biosciences Building, Crown Street, University of Liverpool, Liverpool, L69 7ZB, UK

Table of content

Supplementary figures……S3

Material and methods……S7

Chemical synthesis……S9

References……S13

Spectral data……..S14

Supplementary figures

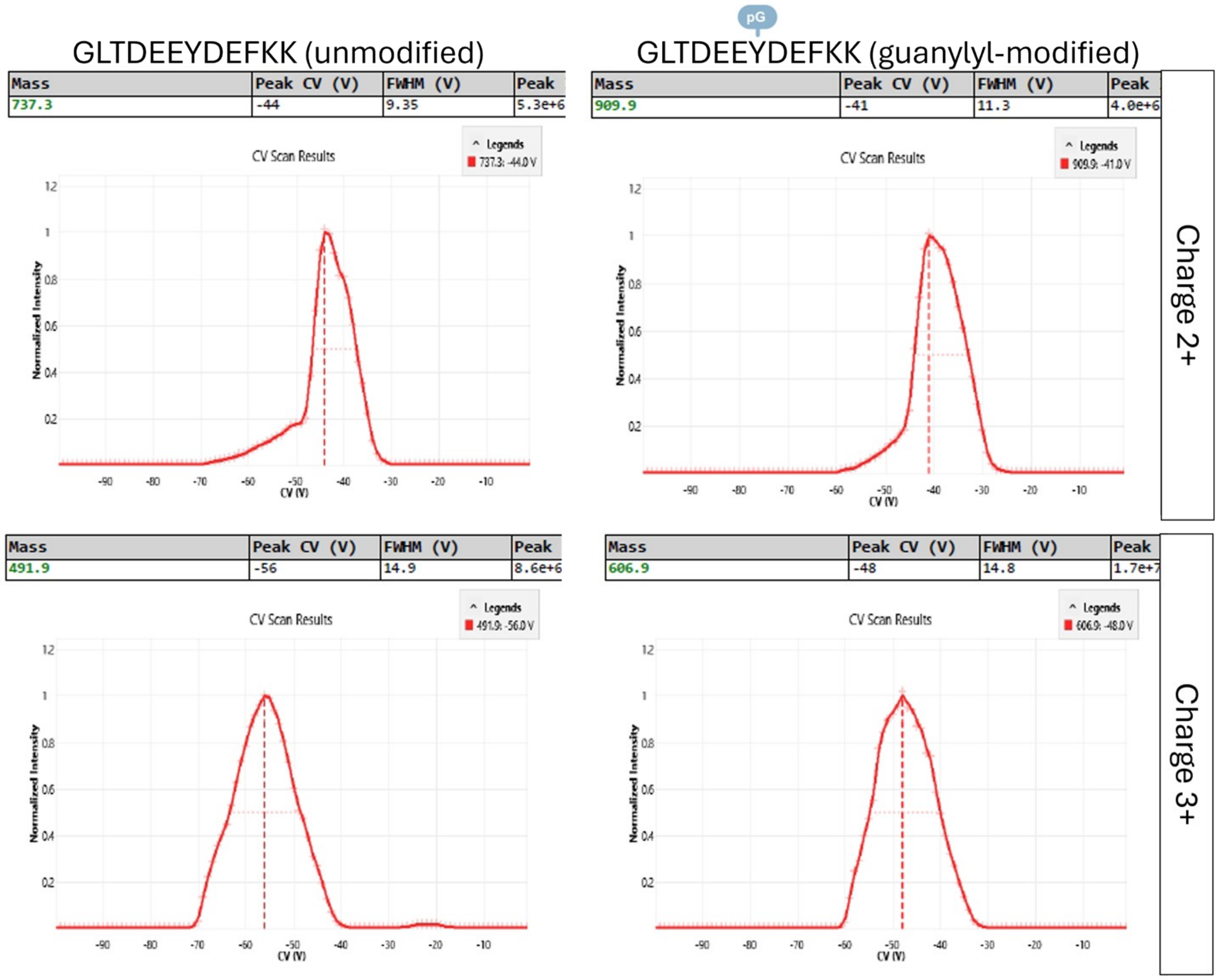

Figure S1. Guanylyl-tyrosine and its effect on the ion-mobility of VPg peptide. Observed peak Compensation Voltage (CV) in a FAIMS Pro system of guanylylated synthetic peptide were 3 to 8 units lower than what was observed with the doubly and triply charged unmodified counterparts, respectively.

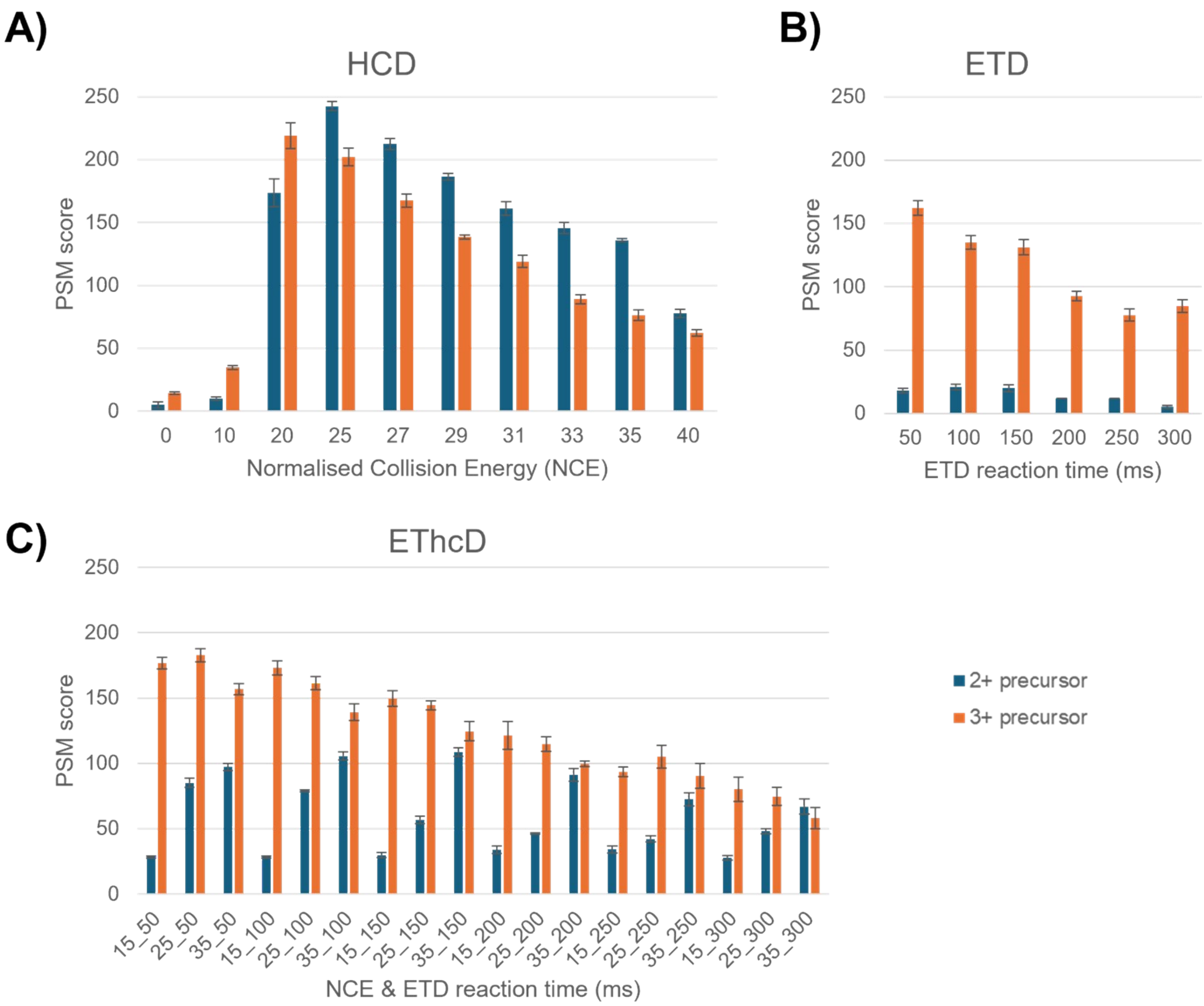

Figure S2. PSM scores versus activation modes and settings. (A) In HCD mode, PSM scores peak at 25% and 20% NCE for 2+ and 3+ precursors, respectively. (B) ETD fragmentation yielded higher scores when applied to 3+ precursors, particularly with short reaction times. (C) Similarly, EThcD fragmentation of 3+ precursors showed higher scores overall, with peak scores around 50 ms ETD reaction time followed by HCD using NCE of 25%. *n = 5,* MS/MS scans.

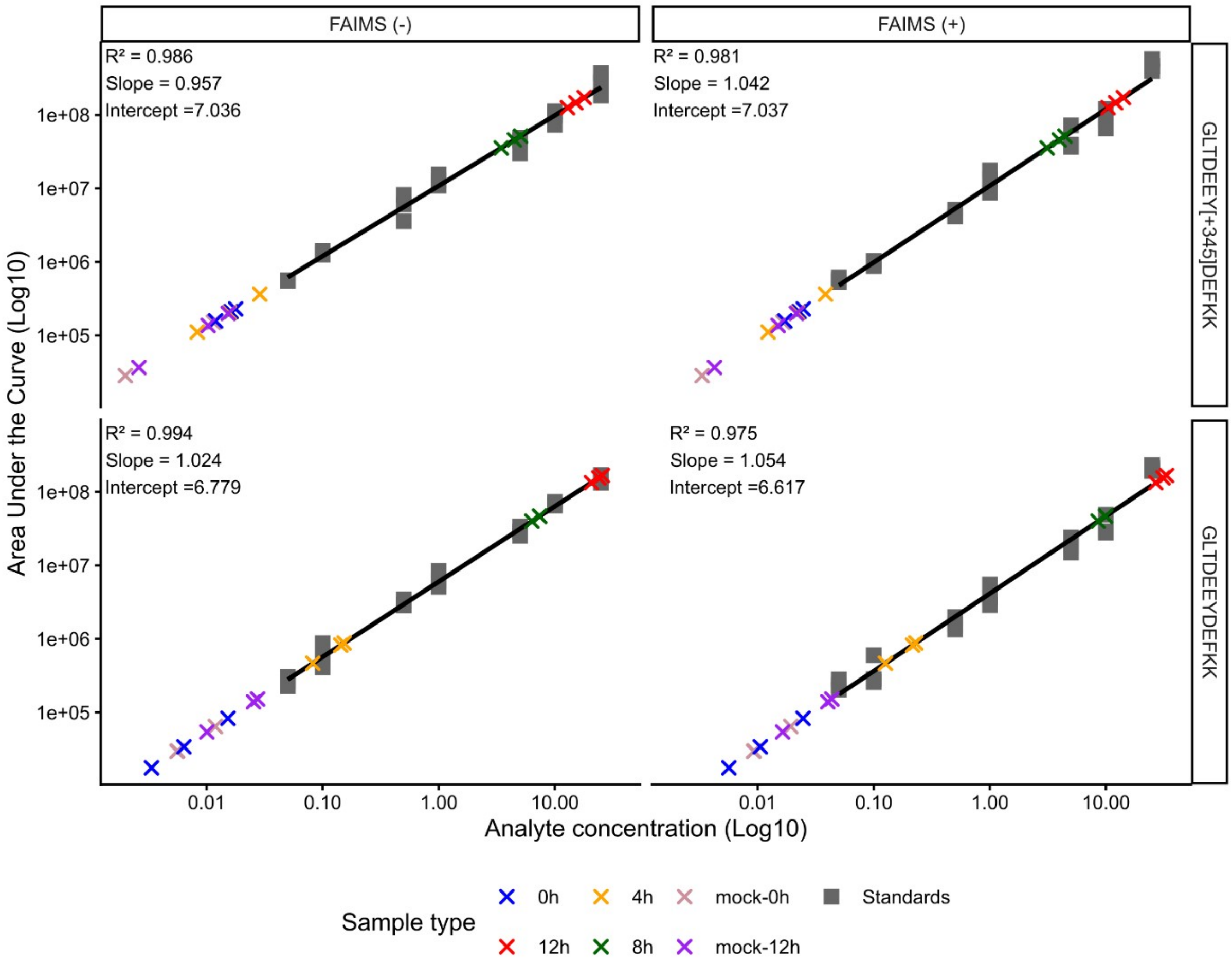

Figure S3. Matrix-matched calibration curve with synthetic VPg peptides. Dilution series from 25 fmol to 50 amol of VPg peptides on-column per 300 ng of mock-infected BV2 cells digest matrix acquired by PRM without or with FAIMS Pro operating with the optimised compensation voltages as per Figure S1. R-squared ranged between 0.97-0.99 indicating a robust linear response within the tested range irrespective of whether FAIMS was enabled or not. *n* = 3, technical replicates. Spiked-in standards denoted by grey squares. For comparison, samples from the time course MNV infection (acquired without FAIMS) were overlaid onto the calibration curves (crosses) confirming both VPg peptides detected within the linear range at 8 and 12 hpi whereas at 4 hpi only the unmodified peptide was consistently detected within the linear range.

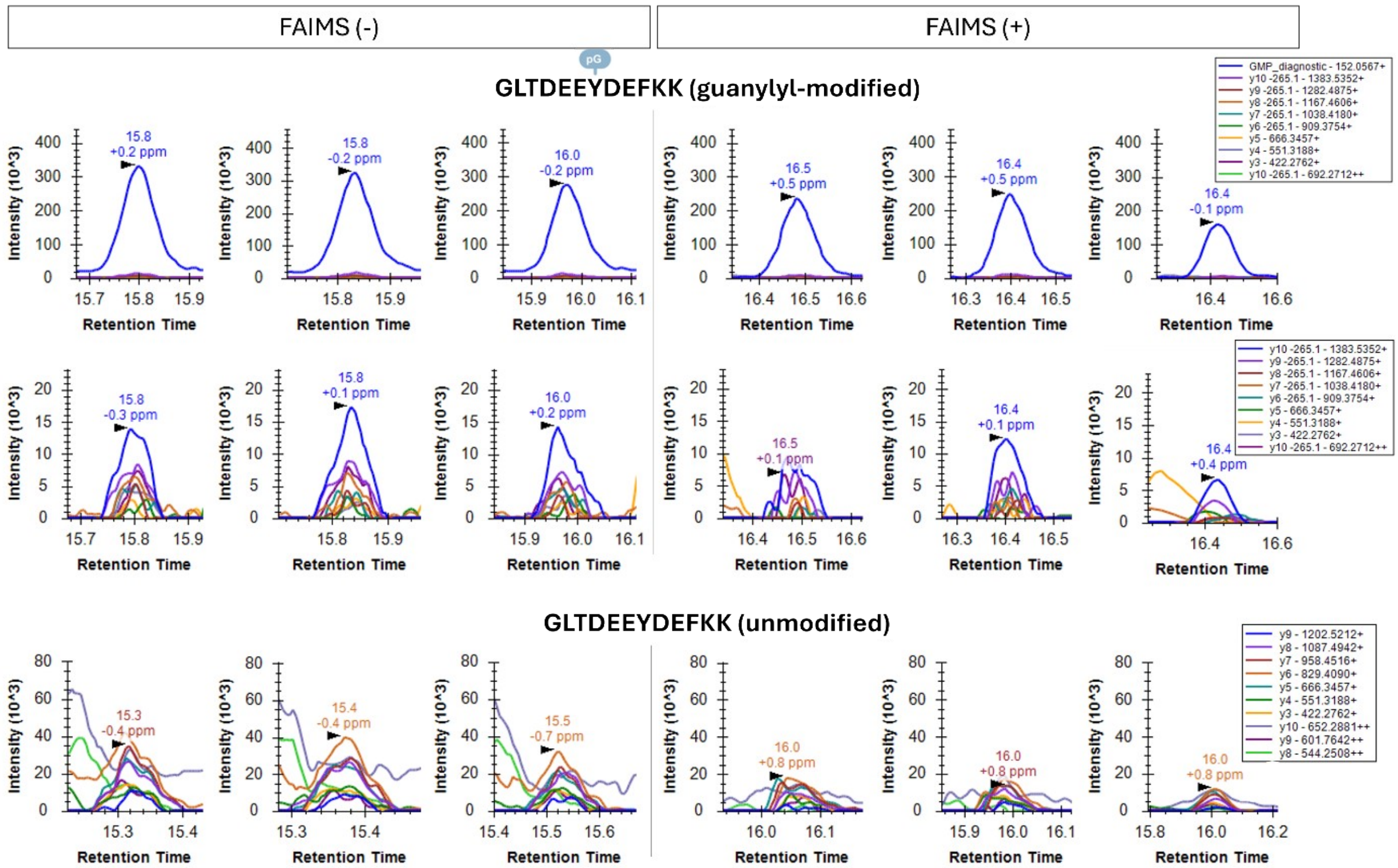

Figure S4. Comparison of synthetic VPg peptide detection with and without FAIMS at the lowest calibration point. Top and middle panels show extracted ion chromatograms (XICs) of the guanylylated VPg peptide with and without the GMP diagnostic ion, respectively. Bottom panel shows XICs of the corresponding unmodified VPg peptide. No improvement in PRM assay sensitivity was observed with the use of FAIMS at the lowest calibration point (approx. 50 amol on-column/300 ng matrix). *n* = 3, technical replicates**.**

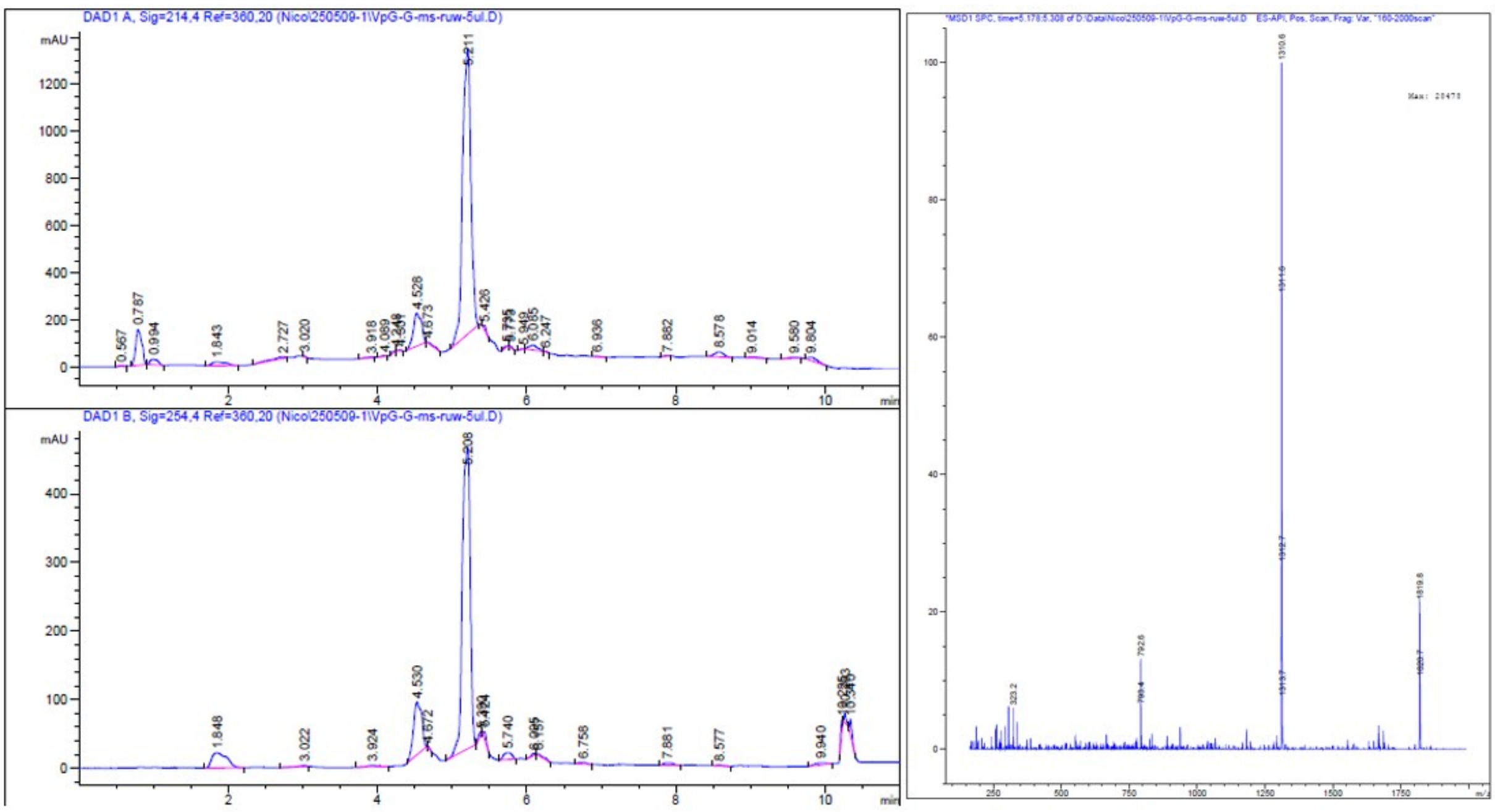

Figure S5. The crude LC-MS trace of the VPg peptide 1, indicating purity.

Material and methods

Murine norovirus infection: A recombinant Murine norovirus 1 (rMNV) was generated as previously described and propagated at low MOI in mouse microglial BV-2 cells.[1] Experimental infections were maintained in Dulbecco's Modified Eagle Medium (DMEM), high glucose, pyruvate, supplemented with 10% FBS and 100 µg/mL penicillin/streptomycin. At 70-80% confluency, BV-2 cells were either mock-infected or infected with rMNV (MOI=3) for 1 hour at 37 ºC, 5% CO_2. After the first hour, the media was changed and the time course was initiated. Cells were harvested at 0h, 4h, 8h and 12h using a cell scrapper, pelleted by centrifugation at 300 x *g*, 5 min, room temperature, and washed twice in DPBS before cell pellets were frozen at –80 ºC.

Sample preparation: Cell pellets were probe sonicated (6 series x 10 sec ON / 20 sec OFF, 30% amplitude) in 50 mM HEPES pH 8.0 containing 1% (w/v) sodium dodecyl sulphate, 1% (w/v) sodium deoxycholate, 1% (v/v) IGEPAL-CA630, 500 mM NaCl and 1X Halt Protease and Phosphatase Inhibitor Cocktail. Protein concentration was determined using Pierce BCA Protein Assay Kit, following the manufacturer's microplate protocol. Lysates were treated with 4 mM dithiothreitol, 60 ºC, 10 min, then cooled down in ice-bath for 2 minutes and then treated with 14 mM iodoacetamide for 30 minutes at room temperature in the dark. At the end, the excess of iodoacetamide was quenched with 3 mM dithiothreitol, 10 min, room temperature. Aliquots containing 15 µg total protein were subjected to SP3 clean-up protocol and overnight MS-grade trypsin digestion (1:50 enzyme to protein ratio) as described elsewhere.[2] Trypsinolysis was terminated by the addition of trifluoroacetic acid at 0.2% (v/v) final concentration.

LC-MS analysis: Initial evaluation of synthetic peptides to determine optimal FAIMS (Field Asymmetric Waveform Ion Mobility Spectrometry) compensation voltage (CV) and fragmentation settings for HCD, ETD and EThcD activation methods was carried out on a Orbitrap Fusion Lumos Tribid mass spectrometer equipped with a FAIMS Pro system (Thermo Scientific, Bremen, Germany). Peptides were reconstituted to 100 femtomol/µL in 50% MeCN : 49.9% H_2O : 0.1 % formic acid (v/v) and analysed via direct injection using a 500 µL Hamilton syringe, at 1 µl/min. The mass spectrometer was operated in positive mode and peptides were ionised using a Nano EASY-Spray bullet type emitter (Thermo Scientific). Optimal FAIMS CV were determined using the CV Scan using Standard Resolution, within a (-)100 to (-)1 range, whilst doubly and triply charged peptide ions were measured via Selected Ion Monitoring (SIM), with a 2.0 Th isolation window and Orbitrap resolution of 120,000 @200 *m/z*. After determining optimal FAIMS cv for the unmodified (491.8963 *m/z*, 3+; 737.3408 *m/z*, 2+) and guanylylated (606.9121 *m/z*, 3+; 909.8645 *m/z*, 2+) peptide ions, MS/MS scans were acquired under increasing Normalised Collision Energy (NCE; 0-40%) for HCD (Higher-energy Collision Dissociation), or increasing ETD reaction time (50-300 ms), or a combination of increasing NCE and ETD reaction times in EThcD mode. Five MS/MS scans were acquired for each tested condition using an Orbitrap resolution of 120,000. Raw files are available via ProteomeXchange with identifier PXD075200.

A Parallel-Reaction Monitoring (PRM) assay was carried out for the relative quantification of both unmodified and guanylylated GLTDEEYDEFKK VPg peptides in samples from an MNV infection time course. Approximately 300 ng of tryptic digest was resolved using a 30SPD gradient in an EVOSEP One (Evosep Biosystems, Odense, Denmark) equipped with an Aurora Elite column (15 cm x 150 µm ID, 1.7 µm C18; IonOptics). Eluting peptides were measured using an Orbitrap Exploris 480 mass spectrometer (Thermo Scientific, Bremen, Germany) operating in positive mode. Survey scans (350-2000 *m/z*) were acquired at 60,000 resolution @200 *m/z*, 300% AGC target. MS/MS scans targeting the triply charged precursors of the unmodified (491.8963, 3+) and modified VPg peptide (606.9121, 3+)

were acquired at a resolution of 60,000 @200 *m/z*, using a 1.4 Th isolation window, 500% AGC target, 500 ms injection time and 30% normalised HCD energy. PRM data are available via ProteomeXchange with identifier PXD075074 and Panorama Public (https://doi.org/10.6069/4zpa-pw40).

Data analysis: Peptide-spectrum matches (PSM) were annotated and scored using FragmentLab v2.8.1.0 (Richard Scheltema Lab) with default settings (20 ppm mass error) and a custom guanylyl (+345.04) modification with a 152.06 *m/z* diagnostic ion and 265.08 *m/z* neutral loss. Annotated MS/MS are openly available in figshare at https://doi.org/10.6084/m9.figshare.31492393. PRM data was analysed using Skyline v25.1.0.237 and R Studio (R v4.2.0). R scripts are available through https://github.com/emmottlab/guanylylation_2026.

Chemical synthesis

General experimental
All chemicals were used as received unless stated otherwise. Molecular sieves were activated by flame drying *in vacuo* before use. Solvents were dried over activated 4 Å molecular sieves except for MeCN, which was dried over 3 Å molecular sieves. Reactions were performed under a N_2 atmosphere unless stated otherwise. Reaction mixtures were concentrated *in vacuo* using rotary evaporators at 40-50 °C unless stated otherwise. Reactions were monitored by thin layer chromatography (TLC) analysis using silica gel 60 F254 coated aluminium sheets from Merck. TLC plates were visualized with ultraviolet light (254 nm) or sprayed with H_2SO_4 (20% v/v in MeOH), potassium permanganate (1 g $KMnO_4$, 5 g K_2CO_3, in 200 ml H_2O) or ceric ammonium molybdate (1 g $Ce(NH_4)_4(SO_4)_4$•$2H_2O$, 2.5 g $(NH_4)_6Mo_7O_{24}$•$4H_2O$, 10 ml H_2SO_4 in 90 ml H_2O). ^{1}H- and ^{13}C NMR spectra were recorded on a Bruker AV-400 or AV-500 NMR. Coupling constants (*J*) are given in Hz. ^{13}C NMR spectra are acquired via the attached proton test (APT) experiment and are presented with even signals (C_q and CH_2) pointing upwards and odd signals (CH and CH_3) pointing downwards. ^{13}C NMR spectra are proton decoupled. The chemical shifts are noted as δ-values in parts per million (ppm) relative to the tetramethylsilane signal (TMS, δ = 0 ppm) or the corresponding solvent signal for ^{1}H NMR and relative to the corresponding solvent signal for ^{13}C NMR. ^{31}P NMR spectra are proton decoupled and the ppm values are indirectly calibrated with H_3PO_4. Structural assignments were made with additional information from gCOSY, gHSQC, and gHMBC experiments. HRMS samples were prepared in either MeCN or milliQ grade H_2O and diluted in a solution of MeCN/milliQ grade H_2O/*t*BuOH (1/1/1) with an approximate concentration of 1 mM and measured on a Thermo Scientific LTQ Orbitrap XL.

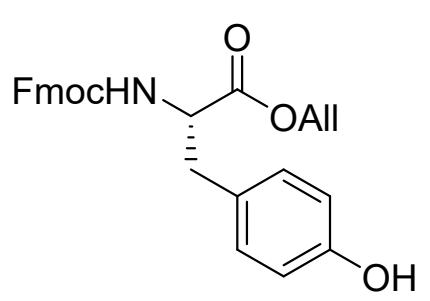

Fmoc-Tyr-OAll (10)
Fmoc-Tyr(O*t*Bu)OH (4.60 g, 10 mmol, 1 eq.) was dissolved in dry DMF (50 mL, 0.2 M) before adding DiPEA (2.1 mL, 12 mmol, 1.2 eq.) and allyl bromide (1.04 mL, 12 mmol, 1.2 eq.). The reaction mixture was stirred overnight at rt. When TLC analysis revealed full conversion, the reaction mixture was quenched with H_2O. The aqueous layer was extracted with Et_2O (3x) and the combined organics were washed with brine (1x). The organics were then dried over Na_2SO_4, filtered and concentrated *in vacuo*. The crude was dissolved in DCM/TFA (20 mL, 0.5 M, 4/1 v/v) and stirred for 4 h until TLC analysis revealed full conversion of the crude starting material. Upon completion, the reaction mixture was concentrated *in vacuo* and co-evaporated with toluene (3x). Column chromatography (pentane/EtOAc, 80/20→70/30) provided **10** (4.18 g, 9.42 mmol, 94%) as a yellowish solid. **^{1}H NMR** (400 MHz, $CDCl_3$) δ 7.76 (d, *J* = 7.6, 1.0 Hz, 2H), 7.56 (dd, *J* = 7.6, 3.1 Hz, 2H), 7.40 (d, *J* = 7.5 Hz, 2H), 7.35 – 7.26 (m, 2H), 6.95 (d, *J* = 8.6 Hz, 2H), 6.72 (d, *J* = 8.5 Hz, 2H), 5.96 – 5.81 (m, 1H), 5.40 – 5.33 (m, 1H), 5.33 – 5.23 (m, 2H), 4.71 – 4.65 (m, 1H), 4.63 (d, *J* = 5.8 Hz, 2H), 4.47 – 4.33 (m, 2H), 4.20 (t, *J* = 7.0 Hz, 1H), 3.05 (m, 2H). **^{13}C NMR** (101 MHz, $CDCl_3$) δ 171.6, 155.9, 155.2, 143.9, 143.7, 141.4, 131.4, 130.6, 127.9, 127.3, 127.2, 125.2, 125.2, 120.1, 119.4, 115.7, 67.2, 66.3, 55.1, 47.2, 37.6. **HRMS** (ESI) m/z: $[M + Na]^+$ calcd for $C_{27}H_{25}NO_5Na^+$ 466.1625, found 466.1625.

***N*²,*N*²-di-*tert*-butoxycarbonyl-6-*O*-*tert*-butyl-2',3'-di-*O*-*tert*-butoxycarbonyl 5'-*O*-*tert*butyldiphenyl silyl guanosine (3)**

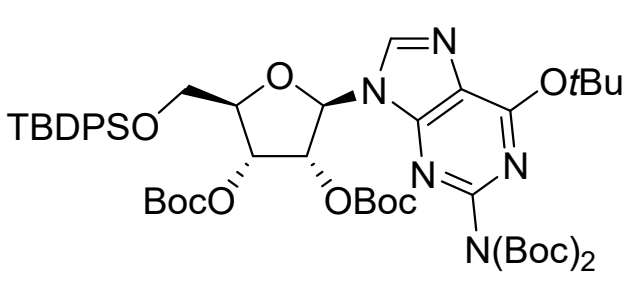

*N*²,*N*²-di-*tert*-butoxycarbonyl-6-*O*-*tert*-butyl guanosine **2** was prepared in three steps according to literature procedures.[3,4] Triol **2** (21.7 g, 40.0 mmol) was dissolved in anhydrous DMF (200 mL, 0.2 M) and imidazole (5.44 g, 80 mmol, 2 eq.) and TBDPS-Cl (11.4 mL, 43.9 mmol, 1.1 eq.) were added. The mixture was stirred at room temperature overnight, followed by quenching with MeOH and concentration under reduced pressure. The crude was re-dissolved in Et_2O and poured into a separatory funnel containing water. The layers were separated and the aqueous

layer was extracted with Et_2O (2x). The pooled organic layers were then washed twice with 1M HCl (aq.), once with $NaHCO_3$ (sat. aq.), once with brine, dried over $MgSO_4$, filtered and concentrated *in vacuo*. The crude 5'-*O*-TBDPS intermediate co-evaporated twice with anhydrous toluene and dissolved in anhydrous DCM (200 mL, 0.2 M). Sequentially, anhydrous Et_3N (dried over KOH pellets; 28 mL, 202 mmol, 5.1 eq.), DMAP (1.46 g, 12 mmol, 0.3 eq.) and Boc_2O (27.5 mL; 120 mmol, 3.0 eq.) were added and the resulting mixture was stirred overnight at room temperature. After TLC(-MS) analysis confirmed complete conversion to the desired compound, the reaction was quenched by the addition of MeOH on ice, after which the mixture was poured over $NaHCO_3$ (sat. aq.). The layers were separated and the aqueous phase was extracted with DCM (3x). The organic layers were combined and dried over $MgSO_4$ and concentrated under reduced pressure. The crude material was purified by column chromatography (pentane/Et_2O, 90/10$\rightarrow$50/50) to yield title compound **3** (31.3 g, 32 mmol, 80% over 2 steps) as an off-white brittle foam. **^{1}H NMR** (400 MHz, $CDCl_3$) δ 8.16 (s, 1H, H), 7.67 (dt, *J* = 8.0, 1.8 Hz, 4H), 7.44 – 7.33 (m, 6H), 6.34 (d, *J* = 6.4 Hz, 1H), 5.62 (dd, *J* = 6.4, 5.1 Hz, 1H), 5.53 (dd, *J* = 5.1, 3.5 Hz, 1H), 4.32 (app. q, *J* = 3.0 Hz, 1H), 3.99 (dd, *J* = 11.8, 2.9 Hz, 1H), 3.83 (dd, *J* = 11.7, 2.9 Hz, 1H), 1.70 (s, 9H), 1.50 (s, 9H), 1.43 (s, 9H), 1.38 (s, 18H), 1.12 (s, 9H). **^{13}C NMR** (101 MHz, $CDCl_3$) δ 161.0, 152.6, 152.3, 151.9, 151.5, 150.7, 140.2, 135.8, 135.5, 132.6, 132.3, 130.1, 130.1, 128.1, 128.1, 121.0, 84.1, 84.1, 83.4, 83.2, 82.9, 82.5, 76.1, 72.9, 63.4, 28.4, 28.0, 27.8, 27.7, 27.1, 19.4.

***N^2,N^2*-di-*tert*-butoxycarbonyl-6-*O*-*tert*-butyl-2',3'-di-*O*-*tert*-butoxycarbonyl guanosine (4)**

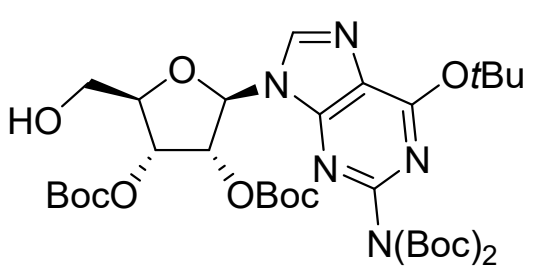

To a solution of protected guanosine **3** (31.3 g, 32 mmol) in anhydrous THF (160 mL, 0.15 M) was added TBAF (1 M in THF; 48 mL, 48 mmol, 1.5 eq.) and the mixture was stirred for 30 min. at room temperature. After TLC analysis confirmed full conversion, the mixture was concentrated to dryness. Column chromatography of the crude material (pentane/EtOAc, 90/10$\rightarrow$64/40) rendered title compound **4** (19.9 g, 26.8 mmol, 84%) as an off-white brittle foam. **^{1}H NMR** (400 MHz, $CDCl_3$) δ 7.92 (s, 1H), 6.01 (d, *J* = 8.0 Hz, 1H), 5.88 (dd, *J* = 8.0, 4.9 Hz, 1H), 5.56 – 5.47 (m, 2H), 4.38 (app. q, *J* = 1.8 Hz, 1H), 3.87 (app. dt, *J* = 12.9, 2.3 Hz, 1H), 3.74 (ddd, *J* = 13.2, 11.2, 2.0 Hz, 1H), 1.69 (s, 9H), 1.50 (s, 9H), 1.38 (s, 18H), 1.36 (s, 9H). **^{13}C NMR** (101 MHz, $CDCl_3$) δ 161.6, 152.4, 151.4, 151.1, 151.1, 150.3, 142.6, 122.8, 87.8, 85.7, 84.8, 83.6, 83.3, 83.2, 74.9, 74.9, 62.7, 28.3, 27.9, 27.8, 27.6. **HRMS** (ESI) m/z: $[M + H]^+$ calcd for $C_{34}H_{54}N_5O_{13}^+$ 740.3713, found 740.3713.

***N^2,N^2*-di-*tert*-butyloxycarbonyl-*6-O-tert*-butyl-2',3'-di-*O*-*tert*-butyloxycarbonyl-5'-*O*-(2-cyanoethoxy)(diisopropylamino)phosphaneyl guanosine (5)**

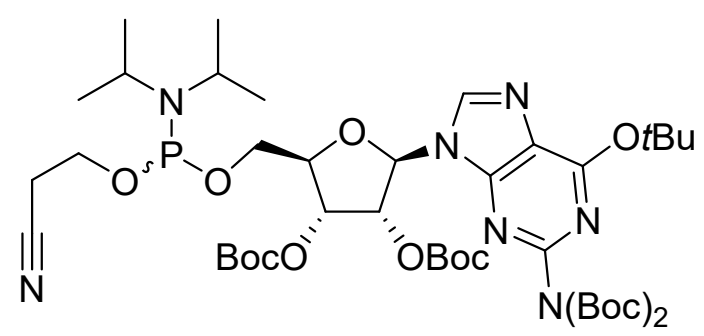

Compound **4** (586 mg, 0.79 mmol, 1 eq.) was co-evaporated with toluene (3x) before dissolving in dry DCM (7.9 mL, 0.1 M). DiPEA (0.41 mL, 2.37 mmol, 3 eq.) and 2-cyanoethyl *N,N*-diisopropylchlorophosphoramidite (0.19 mL, 0.87 mmol, 1.1 eq.) were subsequently added and the reaction mixture was stirred for 4 h at rt until TLC analysis revealed full conversion. Upon completion, the reaction mixture was diluted in DCM and washed with brine (1x). The organic layer was dried over Na_2SO_4, filtered and concentrated *in vacuo*. Column chromatography (pentane/EtOAc/TEA, 90/10/1$\rightarrow$80/20/1) furnished amidite **5** (637 mg, 0.68 mmol, 86% as a mixture of R_p/S_p isomers) as a white foamy solid. **^{1}H NMR** (400 MHz, CD_3CN) δ 8.32 (2x s, 1H), 6.14 (app. t, *J* = 5.6 Hz, 1H), 5.70 – 5.62 (m, 1H), 5.47 – 5.39 (m, 1H), 4.43 – 4.35 (m, 1H), 4.00 – 3.77 (m, 4H), 3.72 – 3.56 (m, 2H), 2.75 – 2.63 (m, 2H), 1.68 (s, 9H), 1.47 (2x s, 9H), 1.44 (s, 18H), 1.38 (2x s, 9H), 1.23 – 1.12 (m, 12H). **^{13}C NMR** (101 MHz, CD_3CN) δ 161.8, 153.5, 153.4, 153.1, 153.1, 152.7, 152.7, 152.2, 152.1, 151.8, 142.5, 142.3, 121.9, 121.8, 86.4, 86.0, 84.8, 84.3, 84.0, 84.0, 83.3, 83.3, 82.9, 82.8, 77.0, 76.8, 74.7, 74.3, 64.1, 64.0, 63.9, 63.8, 59.9, 59.8, 59.6, 59.6, 44.0, 43.9, 43.9, 43.8, 28.6, 28.1, 27.9, 27.7, 25.0, 24.9, 21.0, 20.9. **^{31}P NMR** (162 MHz, CD_3CN) δ 150.57, 150.41. **HRMS** (ESI) m/z: $[M + H]^+$ calcd for $C_{43}H_{71}N_7O_{14}P^+$ 940.4791, found 940.4788.

2-cyanoethoxy-(*N^α*-Fmoc-tyrosin-4-yl allyl ester)-(*N^2,N^2*-di-*tert*-butyloxycarbonyl-6-*O*-*tert*-butyl-2',3'-di-*O*-*tert*-butyloxycarbonyl-β-D-guanosyl-5'-yl) phosphate (6)

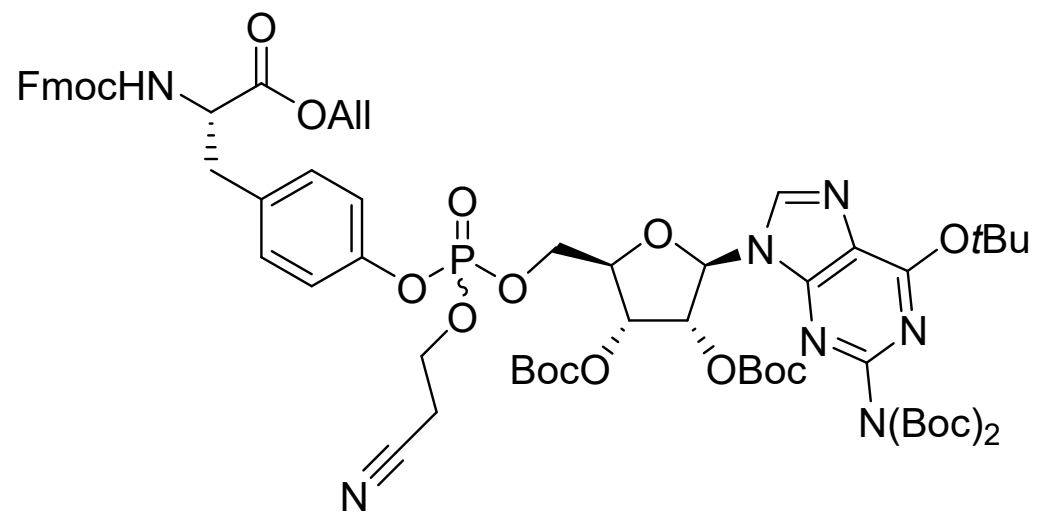

Amidite **5** (637 mg, 0.68 mmol, 1 eq.) and tyrosine **10** (364 mg, 0.82 mmol, 1.2 eq.) were combined and co-evaporated with toluene (3x) before being dissolved in dry MeCN (1.3 mL, final concentration 0.1 M). Freshly activated 3 Å molecular sieves were added and the solution was stirred for 30 minutes at rt. DCI (0.25 M in MeCN, 5.44 mL, 1.36 mmol, 2 eq.) was added to the solution and the mixture was stirred for 30 minutes until ^{31}P NMR revealed full conversion of the phosphoramidite. Upon conversion, *t*BuOOH (5.5 M in decane, 0.49 mL, 2.72 mmol, 4 eq.) was added to the mixture and the solution was stirred at rt for 1h until ^{31}P NMR revealed full conversion to the protected phosphate triester. The reaction mixture was quenched with $NaHCO_3$ (sat. aq.) and extracted with EtOAc (3x). The combined organics were washed with brine (1x), dried over Na_2SO_4, filtered and concentrated *in vacuo*. Column chromatography (DCM/acetone, 98/2→95/5) yielded phosphotriester **6** (734 g, 0.57 mmol, 83% as a mixture of R_p/S_p isomers) as a white foamy solid. **^{1}H NMR** (400 MHz, $CDCl_3$) δ 7.98 (2x s, 1H), 7.79 – 7.71 (m, 2H), 7.60 – 7.52 (m, 2H), 7.43 – 7.34 (m, 2H), 7.33 – 7.27 (m, 2H), 7.19 – 7.12 (m, 2H), 7.07 (d, *J* = 8.3 Hz, 2H), 6.22 (2x d, *J* = 5.7 Hz, 1H), 5.93 – 5.80 (m, 1H), 5.72 – 5.59 (m, 1H), 5.54 – 5.46 (m, 1H), 5.36 – 5.22 (m, 2H), 4.70 – 4.58 (m, 3H), 4.57 – 4.39 (m, 5H), 4.39 – 4.23 (m, 3H), 4.25 – 4.14 (m, 1H), 3.17 – 3.05 (m, 2H), 2.76 – 2.66 (m, 2H), 1.69 (2x s, 9H), 1.48 (2x s, 9H), 1.42 (2x s, 9H), 1.40 (s, 18H). **^{13}C NMR** (101 MHz, $CDCl_3$) δ 171.0, 161.2, 155.8, 152.1, 152.0, 152.0, 151.8, 151.8, 151.5, 151.5, 150.7, 149.2, 149.1, 143.8, 141.4, 133.7, 133.6, 131.4, 131.1, 131.0, 127.8, 127.2, 125.2, 125.2, 121.4, 121.3, 120.3, 120.2, 120.2, 120.2, 120.1, 119.4, 116.2, 85.8, 85.4, 84.3, 84.2, 83.8, 83.7, 83.6, 83.1, 83.1, 80.4, 80.4, 80.3, 75.4, 75.2, 72.5, 72.4, 67.4, 67.2, 67.0, 66.9, 66.3, 63.1, 63.0, 54.8, 47.2, 37.1, 28.4, 28.0, 27.7, 27.6, 19.6, 19.5. **^{31}P NMR** (162 MHz, $CDCl_3$) δ -6.21, -6.36. **HRMS** (ESI) m/z: $[M + H]^+$ calcd for $C_{64}H_{81}N_7O_{20}P^+$ 1298.5269, found 1298.5278.

2-cyanoethoxy-(*N^α*-Fmoc-tyrosin-4-yl)-(*N^2,N^2*-di-*tert*-butyloxycarbonyl-6-*O*-*tert*-butyl-2',3'-di-*O*-*tert*-butyloxycarbonyl-β-D-guanosyl-5'-yl) phosphate (7)

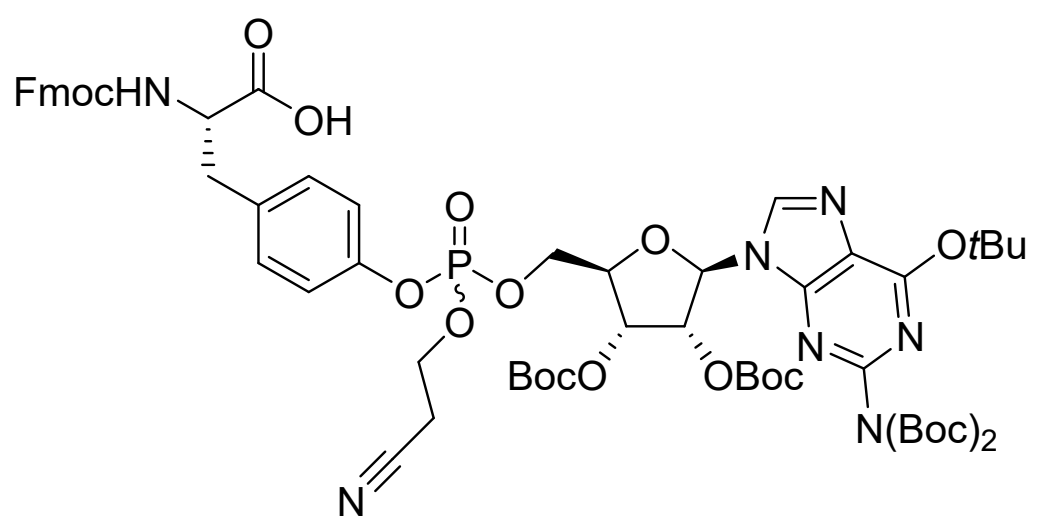

Compound **6** (267 mg, 0.21 mmol, 1 eq.) was dissolved in DCM (2.1 mL, 0.1 M) before adding 1,3-dimethylbarbituric acid (66 mg, 0.42 mmol, 2 eq.) and $Pd(PPh_3)_4$ (23 mg, 0.02 mmol, 0.1 eq.). The reaction mixture was stirred for 4 h at rt. When TLC analysis revealed full conversion, the mixture was diluted in DCM, washed with 1 M HCl (aq., 1x) and brine (1x). The organic layer was dried over Na_2SO_4, filtered and concentrated *in vacuo*. Column chromatography (DCM/MeOH, 99.9/0.01→98/2) provided carboxylic acid **7** (220 mg, 0.17 mmol, 83% as a mixture of R_p/S_p isomers) as a yellow foamy solid. **^{1}H NMR** (400 MHz, MeOD) δ 8.33 (2x s, 1H), 7.76 (d, *J* = 7.5 Hz, 2H), 7.58 (d, *J* = 7.5 Hz, 2H), 7.36 (t, *J* = 7.5 Hz, 2H), 7.32 – 7.23 (m, 2H), 7.24 – 7.15 (m, 2H), 7.16 – 7.07 (m, 2H), 6.24 (2x d, *J* = 4.8 Hz, 1H), 5.83 – 5.73 (m, 1H), 5.61 (q, *J* = 5.4 Hz, 1H), 4.57 – 4.40 (m, 3H), 4.39 – 4.25 (m, 4H), 4.24 – 4.15 (m, 1H), 4.12 (t, *J* = 6.8 Hz, 1H), 3.35 (s, 1H), 3.19 (dd, *J* = 13.6, 4.3 Hz, 1H), 2.97 – 2.87 (m, 1H), 2.87 – 2.75 (m, 2H), 1.71 (2x s, 9H), 1.47 (2x s, 9H), 1.42 (2x s, 27H). **^{13}C NMR** (101 MHz, MeOD) δ 162.3, 158.2, 153.5, 153.4, 153.0, 152.5, 152.2, 150.2, 145.2, 142.5, 136.9, 132.0, 128.8, 128.2, 126.3, 126.2, 122.5, 120.9, 118.2, 88.0, 85.7, 84.8, 84.6, 84.3, 81.6, 81.5, 76.6, 76.5, 73.6, 68.8, 67.8, 65.0, 64.9, 57.2, 37.9, 28.7, 28.2, 28.0, 27.9, 20.1, 20.0. **^{31}P NMR** (162 MHz, MeOD) δ -5.90, -6.02. **HRMS** (ESI) m/z: $[M + H]^+$ calcd for $C_{61}H_{77}N_7O_{20}P^+$ 1258.4956, found 1258.4967.

VPgpG, H-Gly-Leu-Thr-Asp-Glu-Glu-Tyr(pG)-Asp-Glu-Phe-Lys-Lys-OH (1)

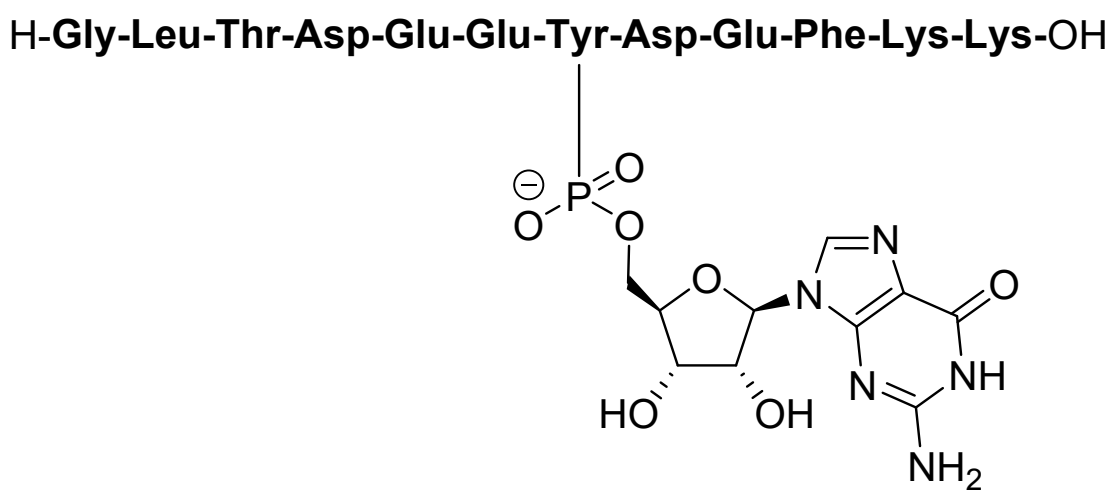

The protected peptide **8** was prepared with a Liberty Blue peptide synthesizer via Fmoc-based solid-phase peptide synthesis, starting from preloaded lysine S PHB TentaGel resin (238 mg, 50 µmol). As a repetitive cycle, 1) three consecutive times Fmoc cleavage DBU in DMF (20 v/v%) at rt. 2) coupling of the indicated amino acid (5 eq.) with activator DIC (5 eq.) and OxymaPure (5 eq.) in DMF (0.1M) at 50°C. The guanylylated tyrosine **7** was introduced using 3 eq. for 2 h at room temperature. The last six amino acids were coupled at room temperature for 2 h as well and the Fmoc group was cleaved with DBU in DMF (20 v/v%) at rt. Acidolysis was achieved with 95% TFA, 2.5% H_2O and 2.5% TIS for 2 h at room temperature. The solution was poured into cold Et_2O, and the precipitate was separated from the supernatant by centrifuging and filtration. The precipitate was concentrated *in vacuo* and purified by RP-HPLC C18. The fractions were collected and lyophilized to obtain guanylylated peptide **1** as white solid (4.7 mg, 2.2 µmol, 4%). **HRMS** (ESI) m/z: $[M + H]^{3+}$ calcd for $C_{75}H_{111}N_{19}O_{32}P^{3+}$ 606.9121, found 606.9130.

References

(1) Chaudhry, Y.; Skinner, M. A.; Goodfellow, I. G. Recovery of Genetically Defined Murine Norovirus in Tissue Culture by Using a Fowlpox Virus Expressing T7 RNA Polymerase. *Journal of General Virology* **2007**, *88* (8), 2091–2100. https://doi.org/10.1099/vir.0.82940-0.

(2) Hughes, C. S.; Moggridge, S.; Müller, T.; Sorensen, P. H.; Morin, G. B.; Krijgsveld, J. Single-Pot, Solid-Phase-Enhanced Sample Preparation for Proteomics Experiments. *Nat. Protoc.* **2019**, *14* (1), 68–85. https://doi.org/10.1038/s41596-018-0082-x.

(3) Simeone, L.; Milano, D.; De Napoli, L.; Irace, C.; Di Pascale, A.; Boccalon, M.; Tecilla, P.; Montesarchio, D. Design, Synthesis and Characterisation of Guanosine-Based Amphiphiles. *Chemistry – A European Journal* **2011**, *17* (49), 13854–13865. https://doi.org/https://doi.org/10.1002/chem.201101827.

(4) Steneker, R.; Rijpkema, K. J.; Ofman, T. P.; Sagiroglugil, M.; Rovira, C.; van der Marel, G. A.; Filippov, D. V; Overkleeft, H. S.; Codée, J. D. C. Modular Synthesis of Nucleoside Diphosphate Sugar Analogues Using Chlorosulfonyl Isocyanate and Dichlorophosphoryl Isocyanate. *Org. Lett.* **2026**. https://doi.org/10.1021/acs.orglett.5c04568.

Spectral data

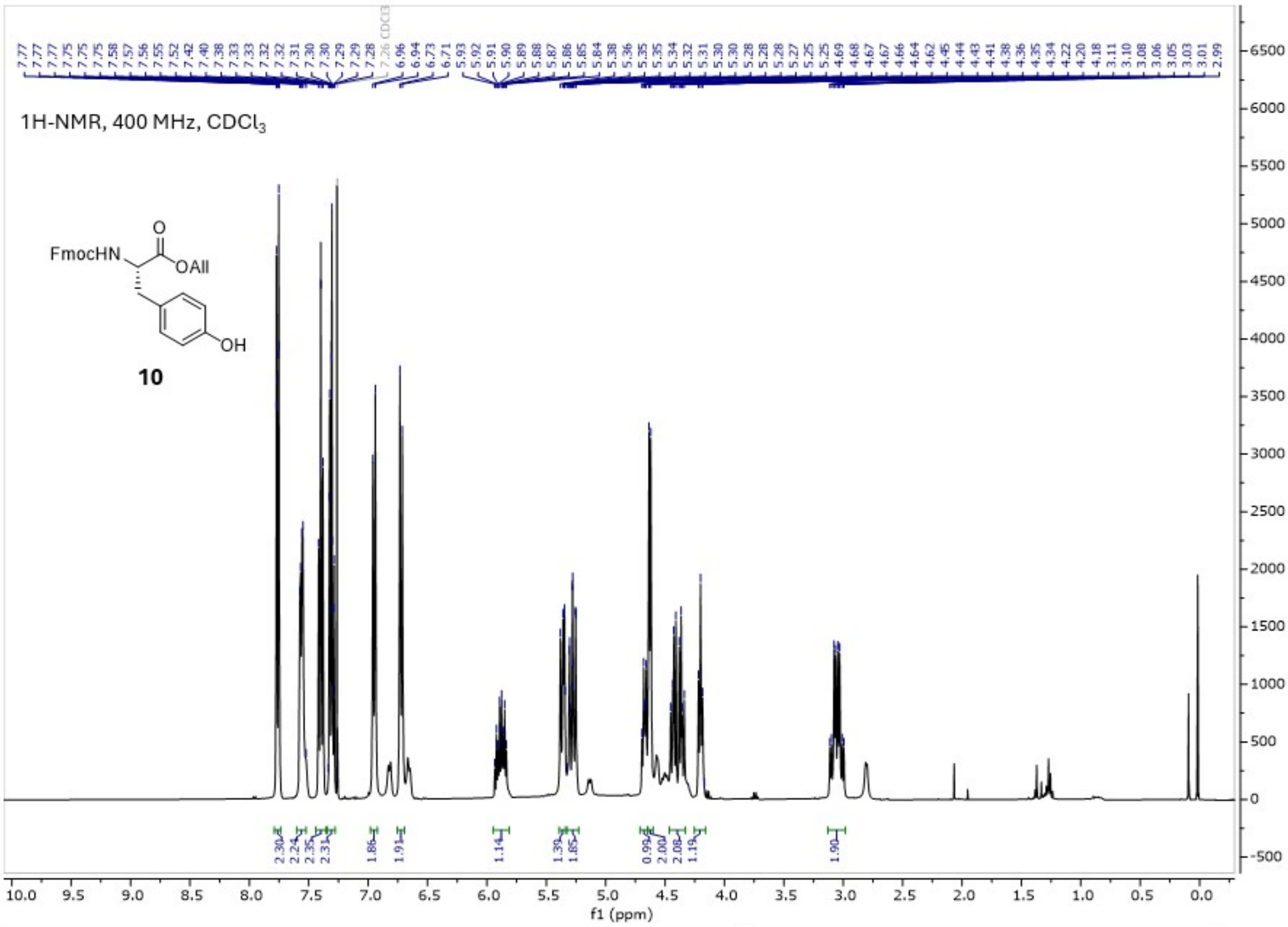

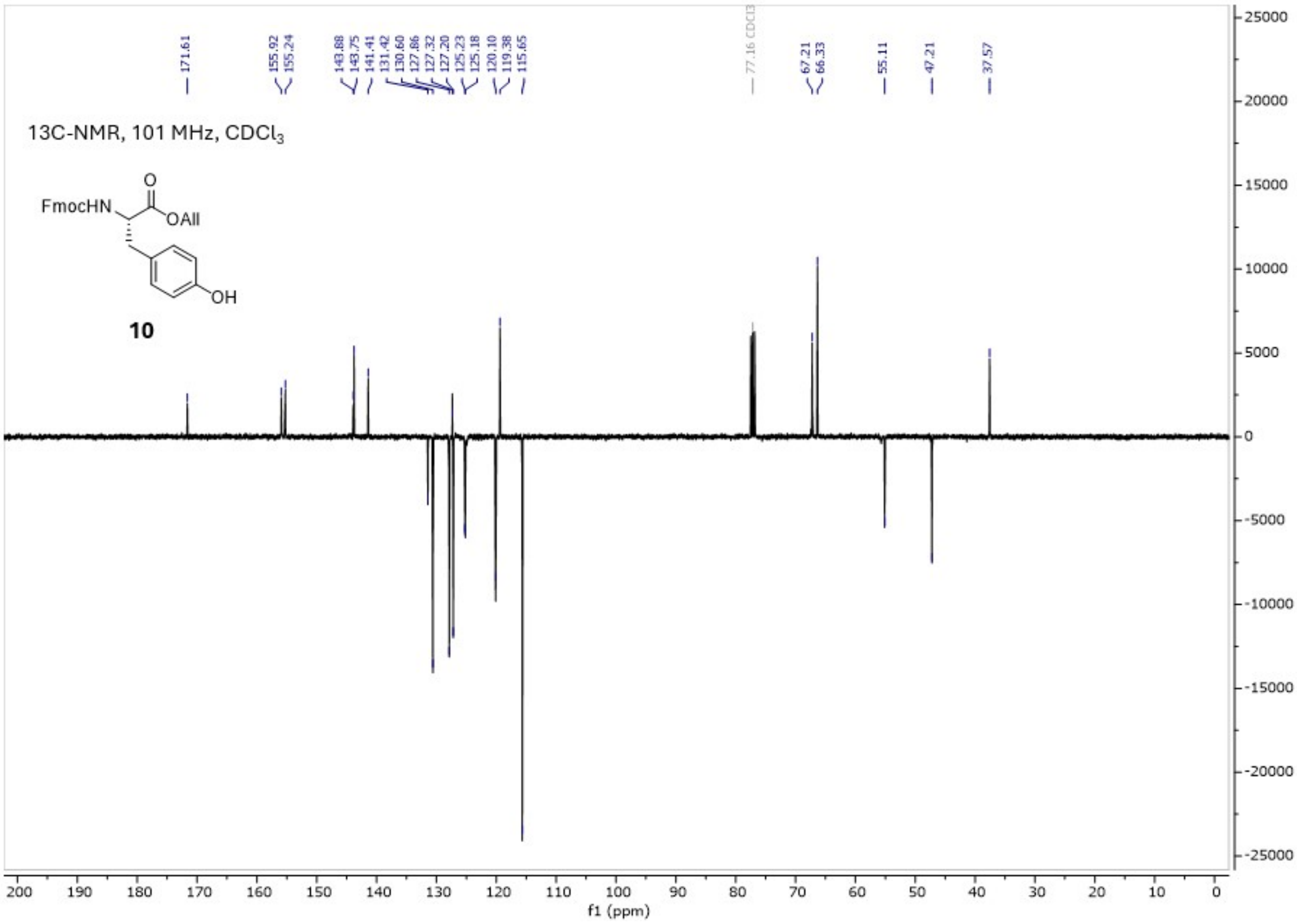

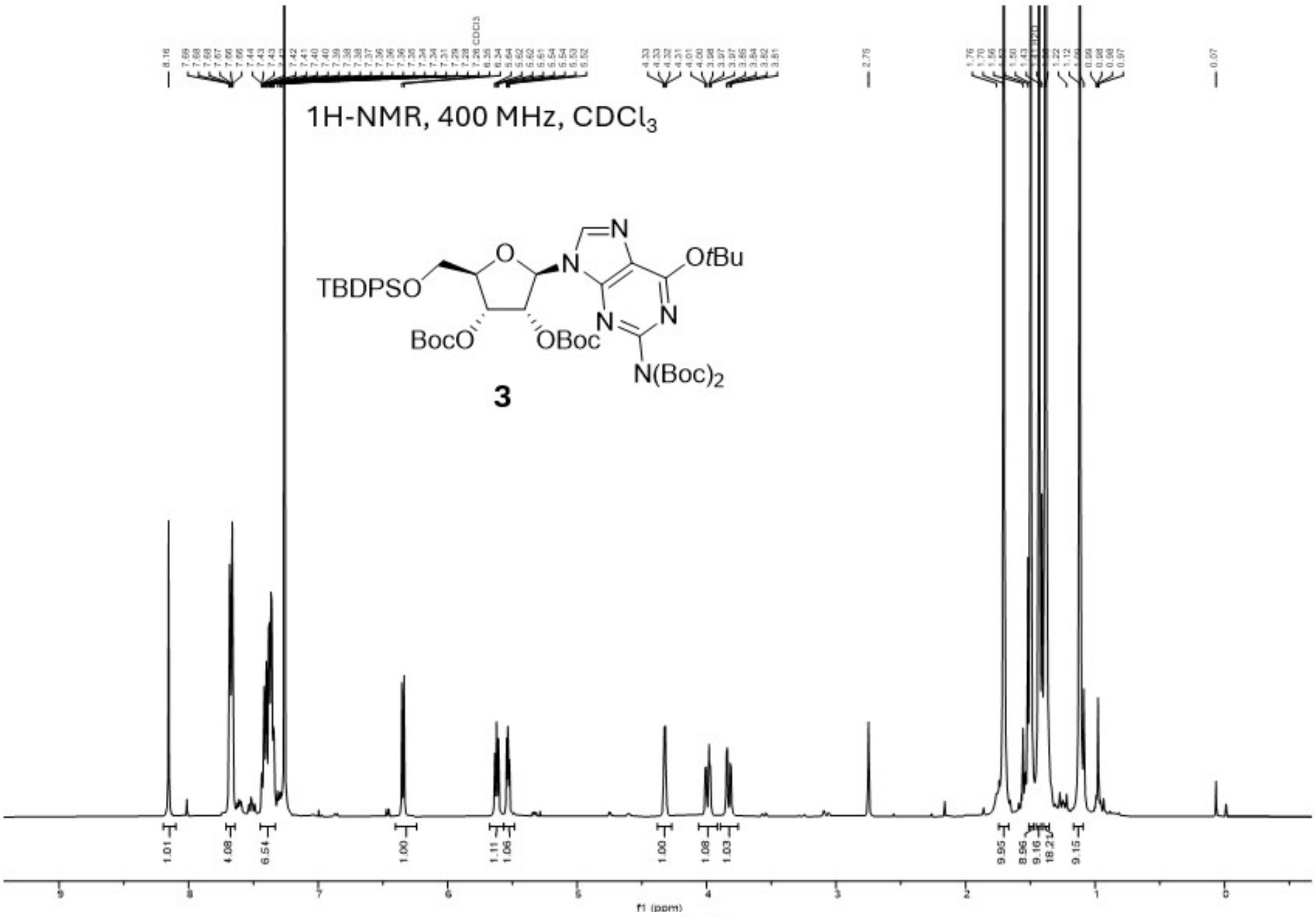
1H-NMR, 400 MHz, CDCl3
TBDPSO
BocO
OBoc
OtBu
N(Boc)2
3
f1 (ppm)

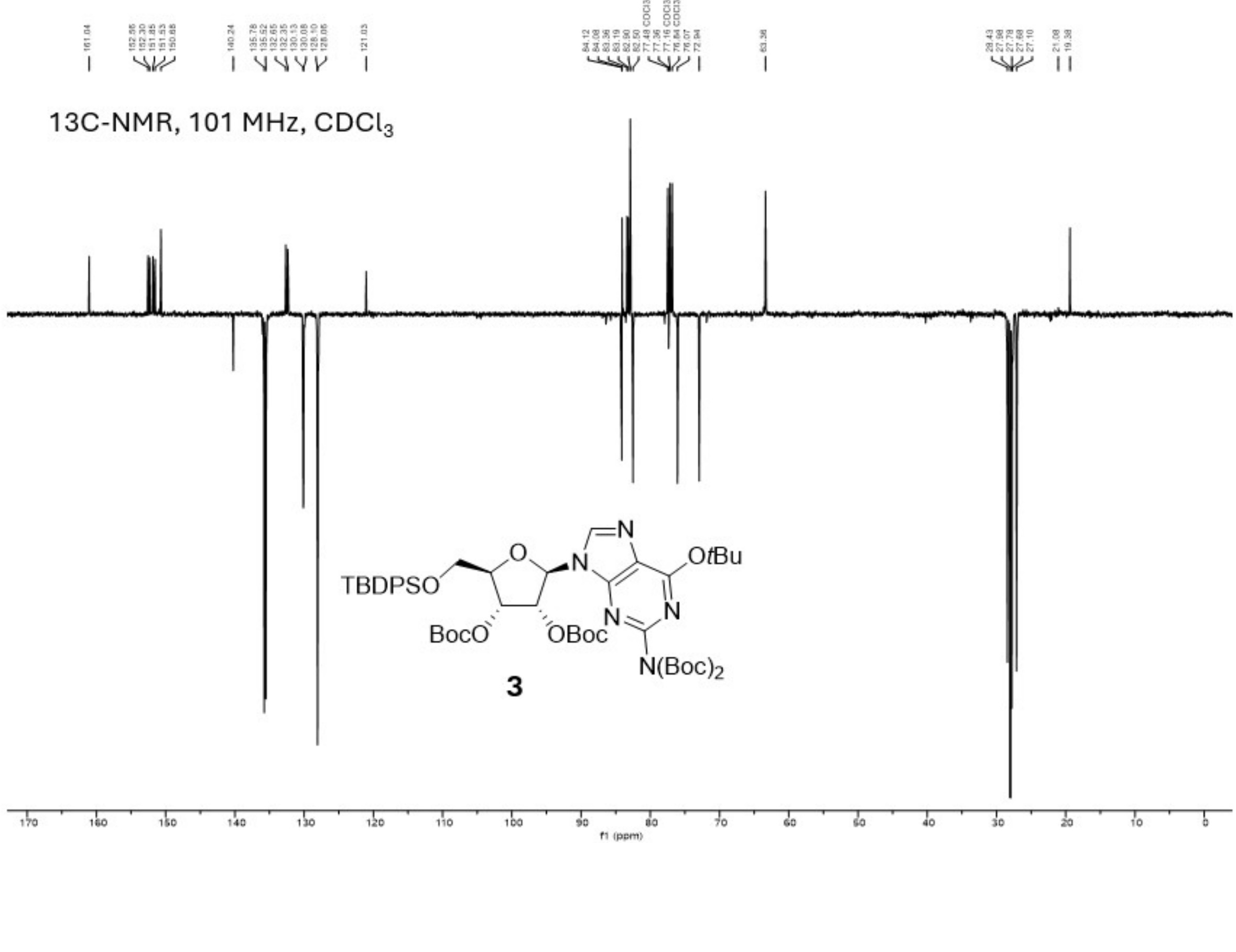
13C-NMR, 101 MHz, CDCl3
TBDPSO
BocO
OBoc
OtBu
N(Boc)2
3
f1 (ppm)

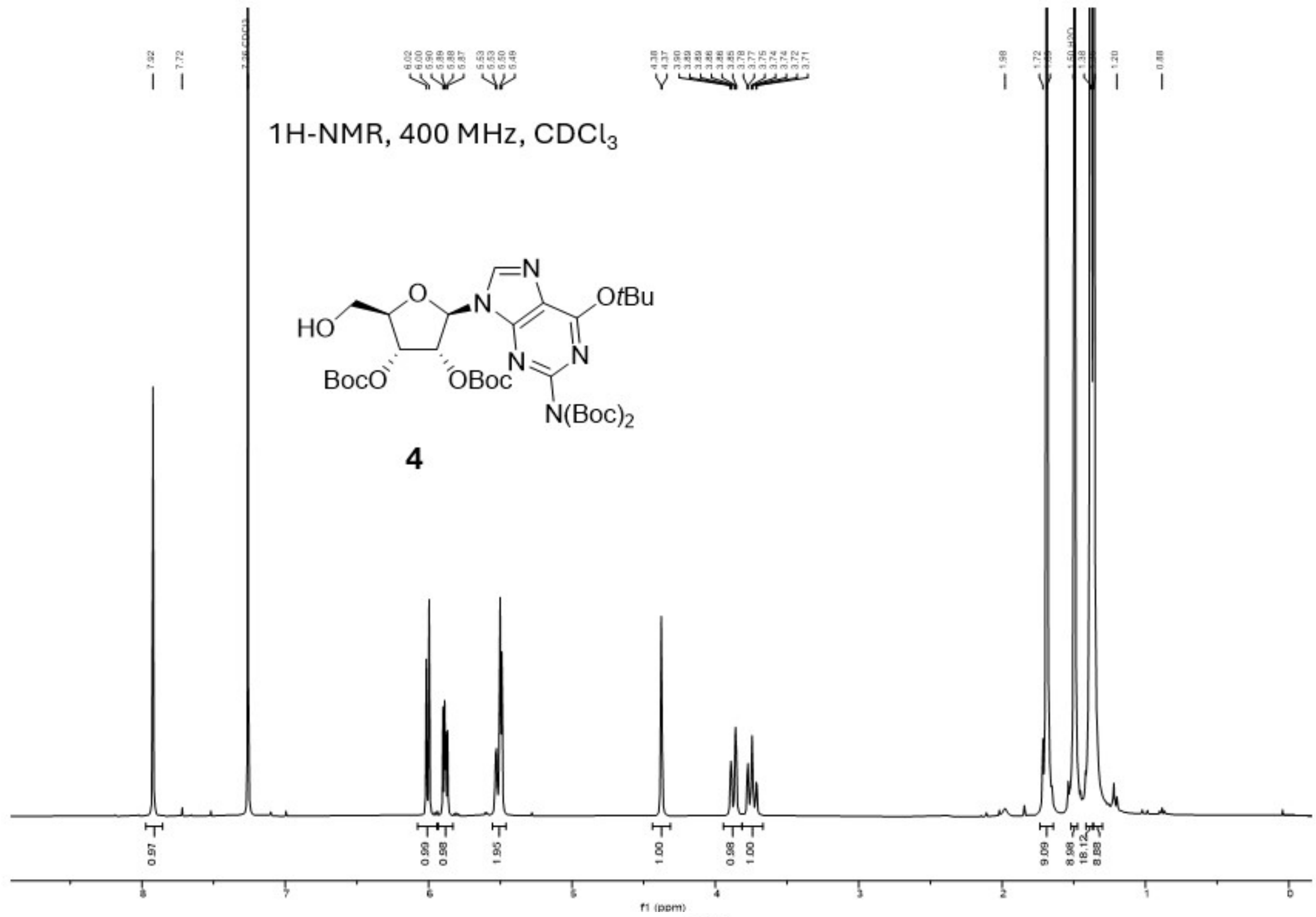
1H-NMR, 400 MHz, CDCl3
HO
BocO
OBoc
OtBu
N(Boc)2
4
f1 (ppm)

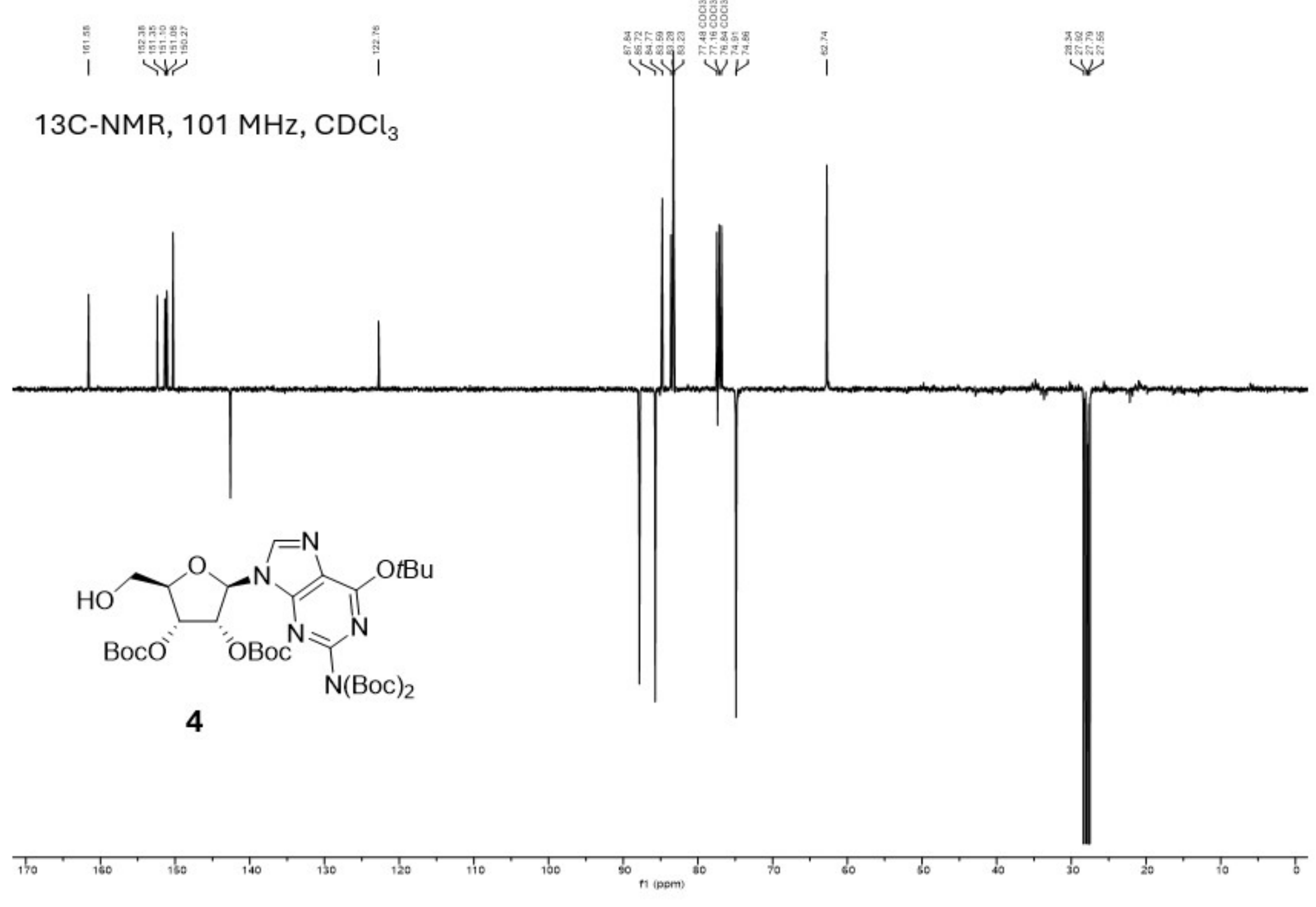
13C-NMR, 101 MHz, CDCl3
HO
BocO
OBoc
OtBu
N(Boc)2
4
f1 (ppm)

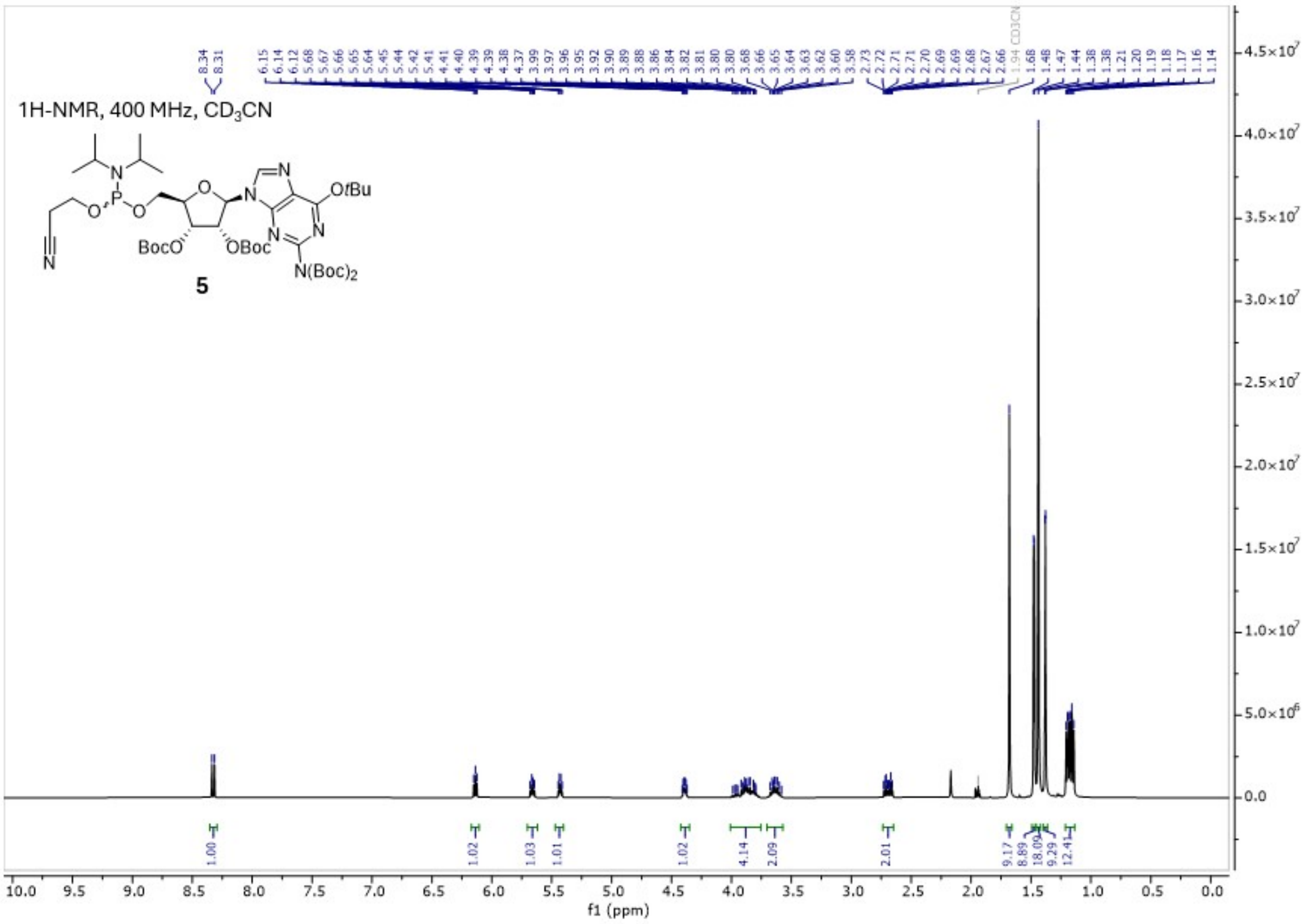
1H-NMR, 400 MHz, CD3CN
OtBu
BocO
OBoc
N(Boc)2
5
8.34 8.31
6.15 6.14 6.12 5.68 5.67 5.66 5.65 5.64 5.45 5.44 5.42 5.41 4.41 4.40 4.39 4.39 4.38 4.37 3.99 3.97 3.96 3.95 3.92 3.90 3.89 3.88 3.86 3.84 3.82 3.81 3.80 3.80 3.68 3.66 3.65 3.64 3.63 3.62 3.60 3.58
2.73 2.72 2.71 2.71 2.70 2.69 2.69 2.68 2.67 2.66
1.94 CD3CN
1.68 1.48 1.47 1.44 1.38 1.38 1.21 1.20 1.19 1.18 1.17 1.16 1.14
1.00 1.02 1.03 1.01 1.02 4.14 2.09 2.01 9.17 8.89 18.09 9.29 12.41
f1 (ppm)
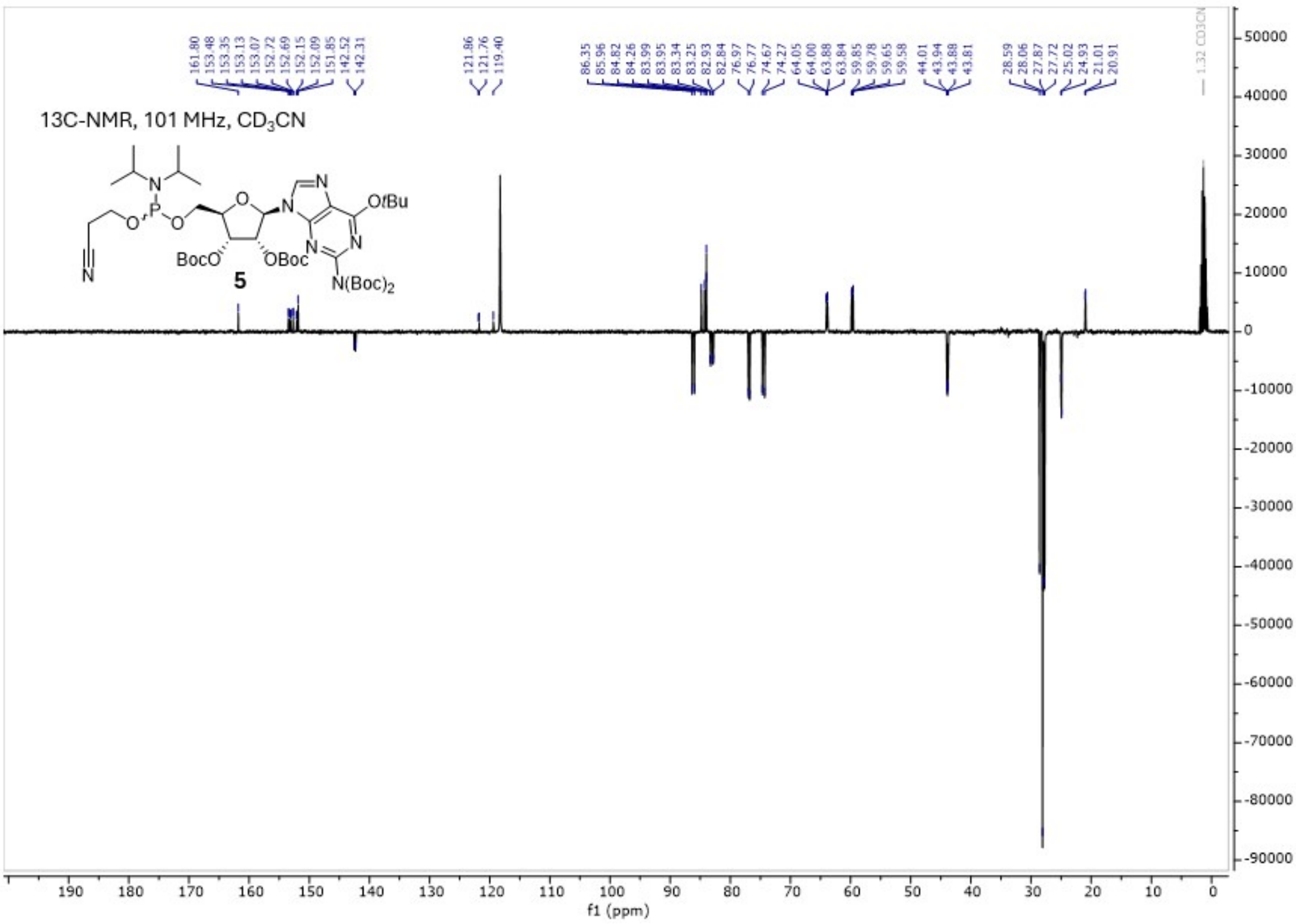
13C-NMR, 101 MHz, CD3CN
OtBu
BocO
OBoc
N(Boc)2
5
161.80 153.48 153.35 153.13 153.07 152.72 152.69 152.15 152.09 151.85 142.52 142.31
121.86 121.76 119.40
86.35 85.96 84.82 84.26 83.99 83.95 83.34 83.25 82.93 82.84 76.97 76.77 74.67 74.27 64.05 64.00 63.88 63.84 59.85 59.78 59.65 59.58
44.01 43.94 43.88 43.81
28.59 28.06 27.87 27.72 25.02 24.93 21.01 20.91
1.32 CD3CN
f1 (ppm)

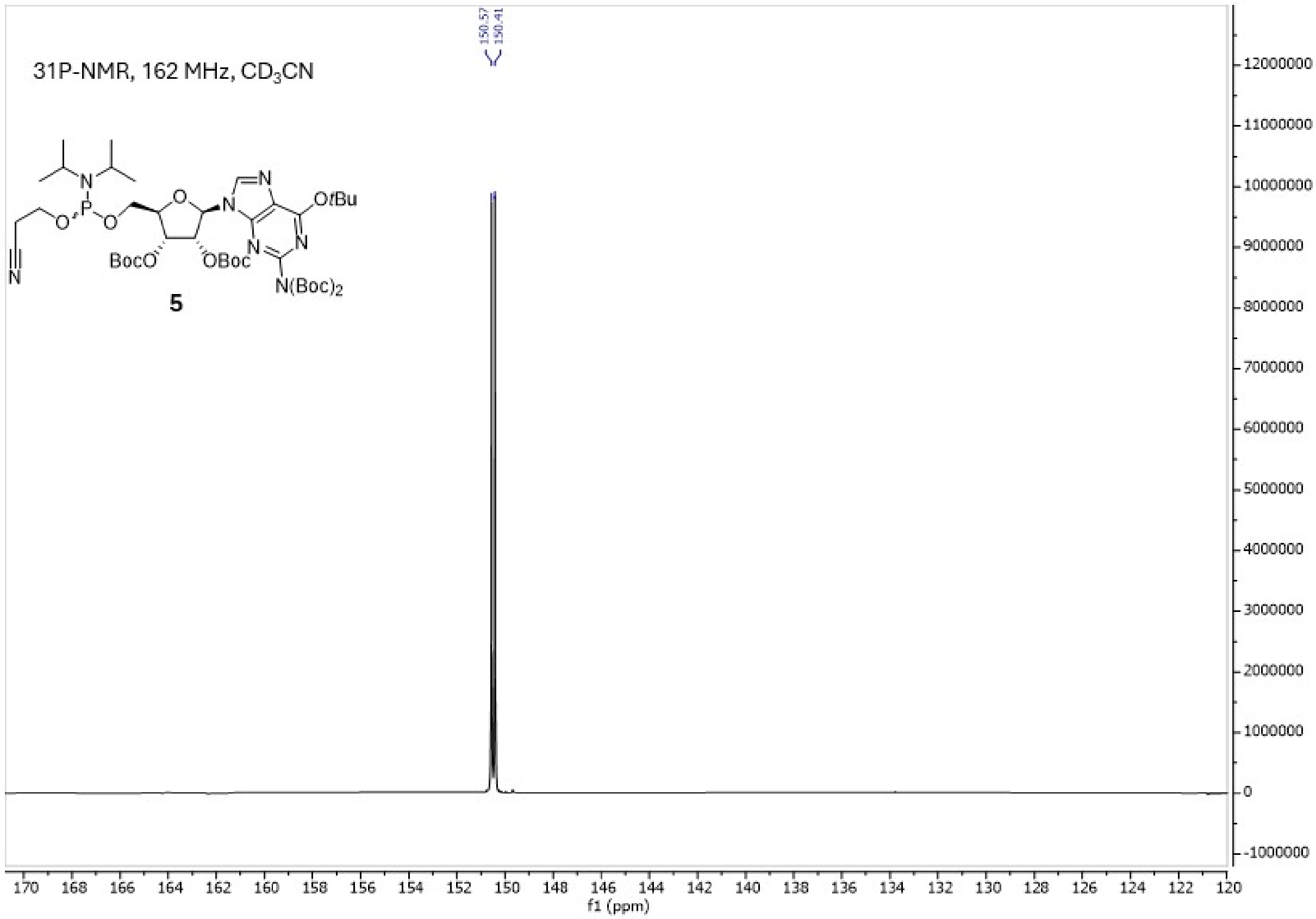
31P-NMR, 162 MHz, CD3CN
5
f1 (ppm)

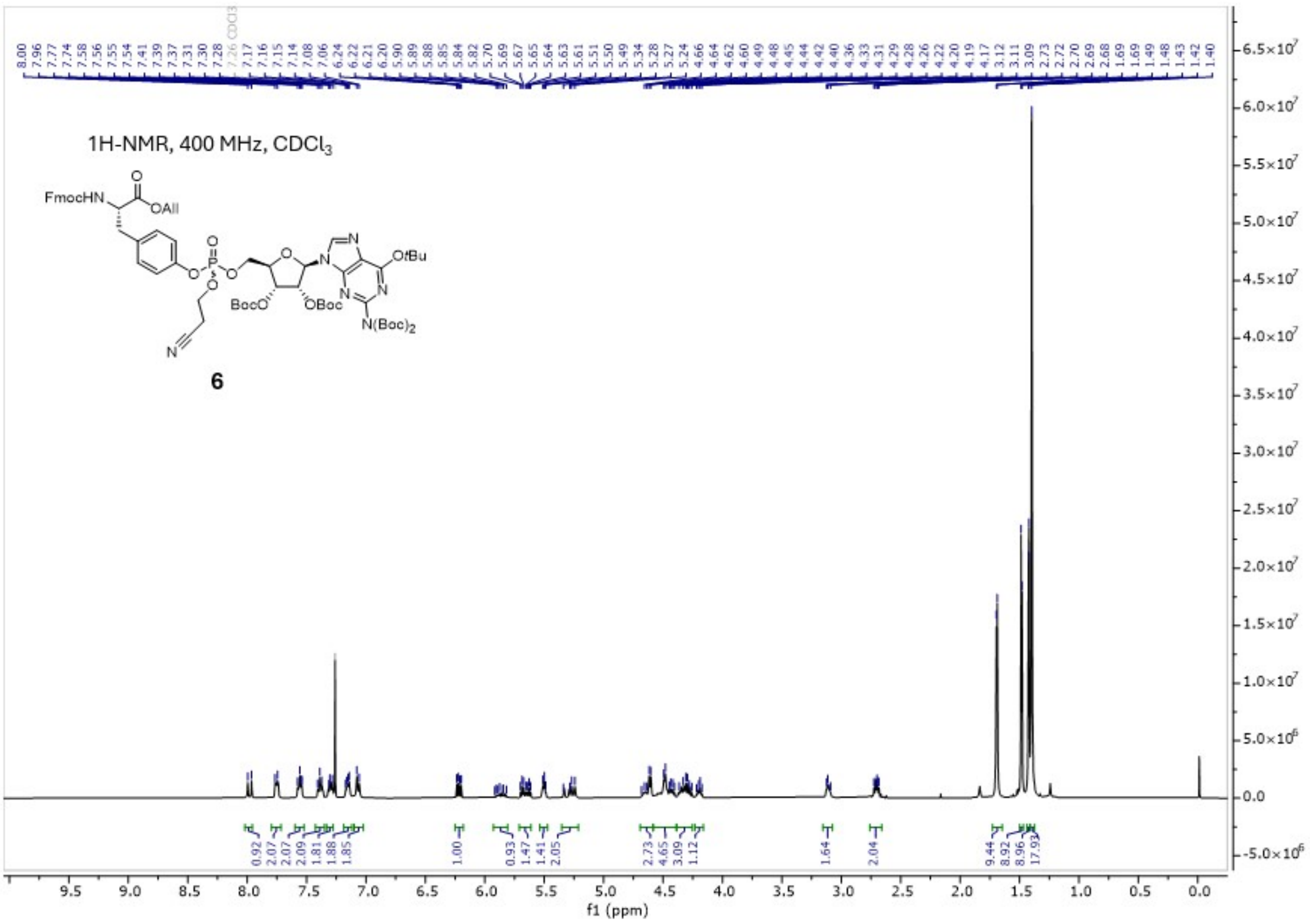
1H-NMR, 400 MHz, CDCl3
6
f1 (ppm)

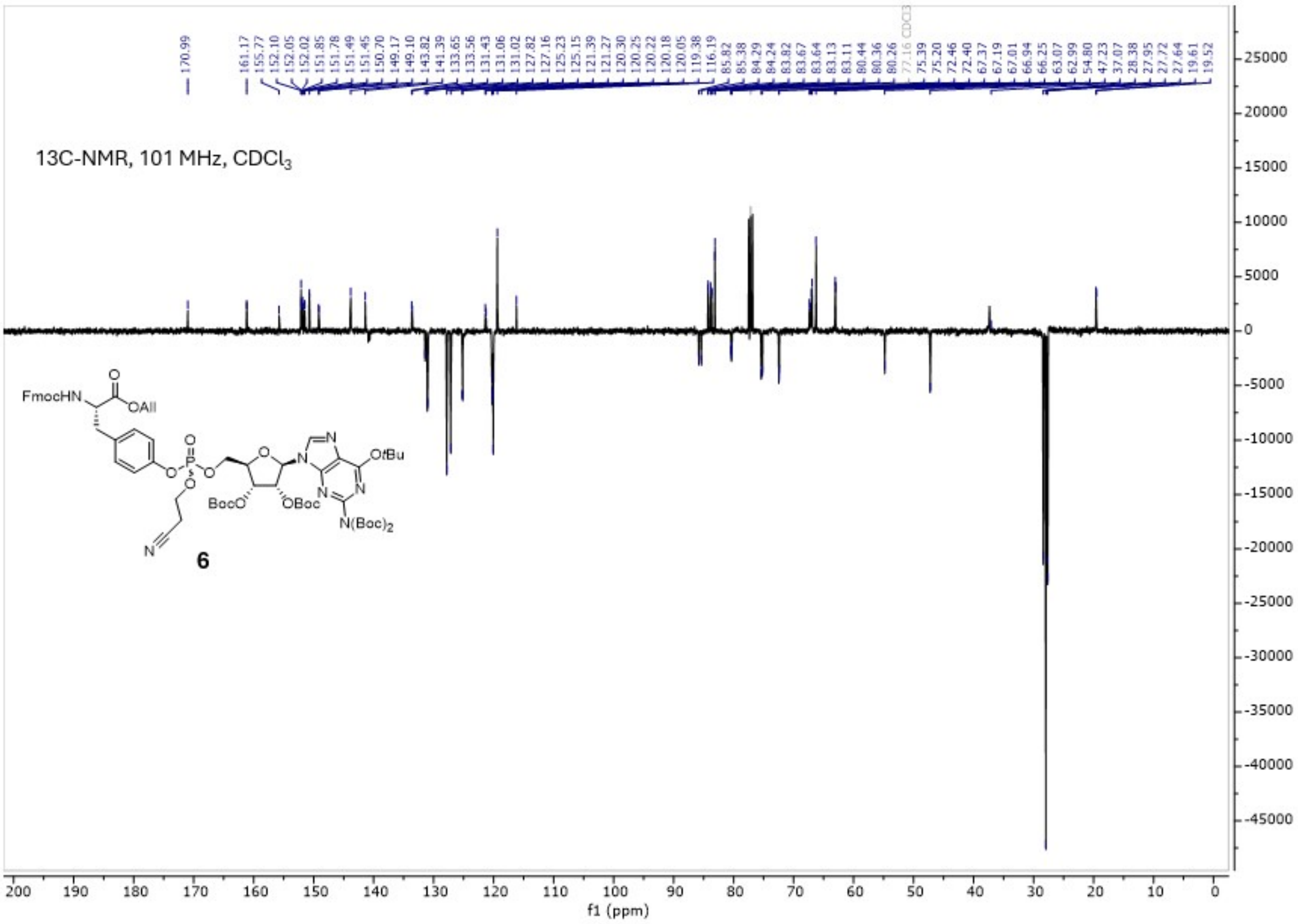
13C-NMR, 101 MHz, CDCl3
170.99
161.17
155.77
152.10
152.05
152.02
151.85
151.78
151.49
151.45
150.70
149.17
149.10
143.82
141.39
133.65
133.56
131.43
131.06
131.02
127.82
127.16
125.23
125.15
121.39
121.27
120.30
120.25
120.22
120.18
120.05
119.38
116.19
85.82
85.38
84.29
84.24
83.82
83.67
83.64
83.13
83.11
80.44
80.36
80.26
77.16 CDCl3
75.39
75.20
72.46
72.40
67.37
67.19
67.01
66.94
66.25
63.07
62.99
54.80
47.23
37.07
28.38
27.95
27.72
27.64
19.61
19.52
FmocHN
OAll
OtBu
BocO
OBoc
N(Boc)2
6
f1 (ppm)

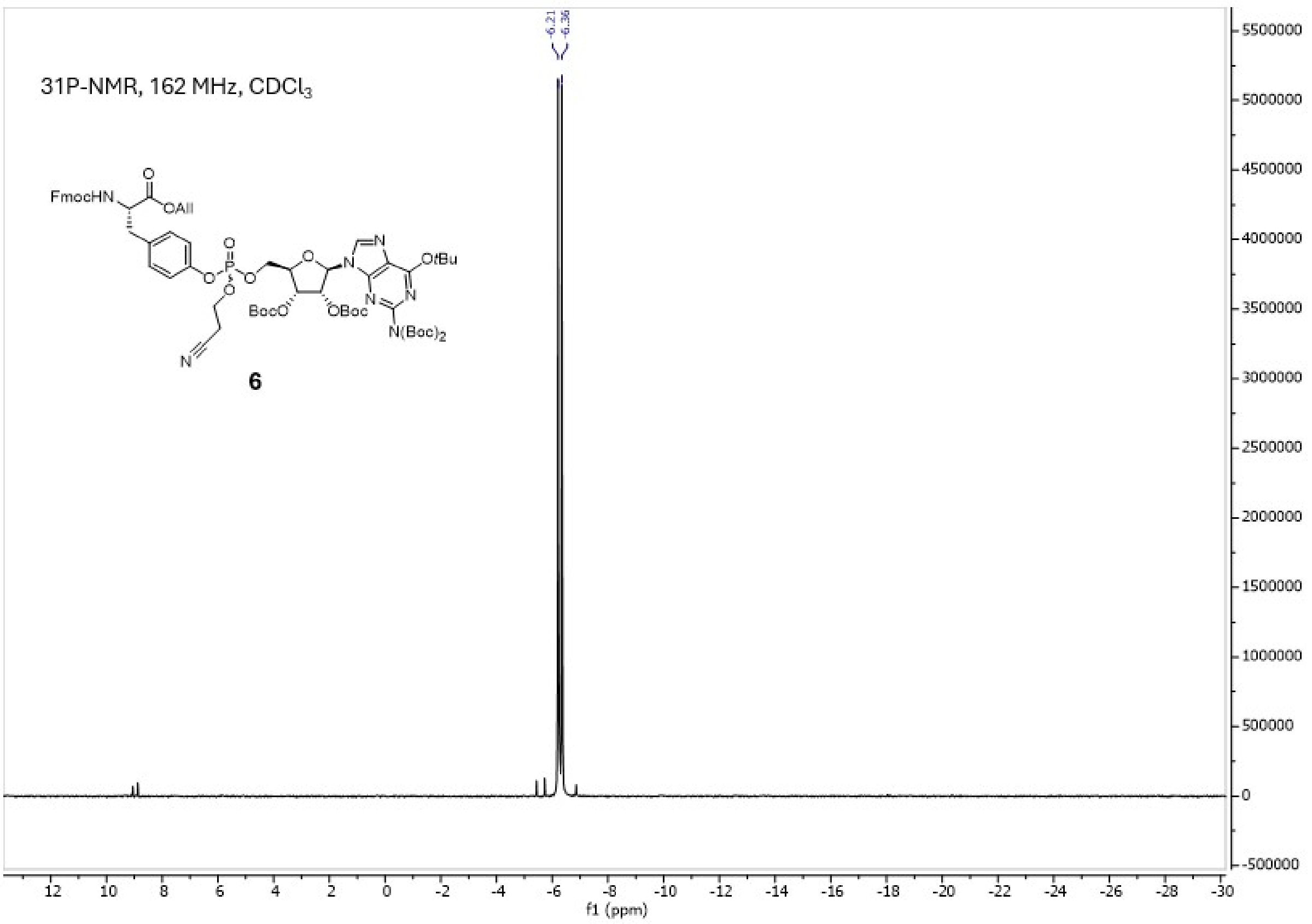
31P-NMR, 162 MHz, CDCl3
-6.21
-6.36
FmocHN
OAll
OtBu
BocO
OBoc
N(Boc)2
6
f1 (ppm)

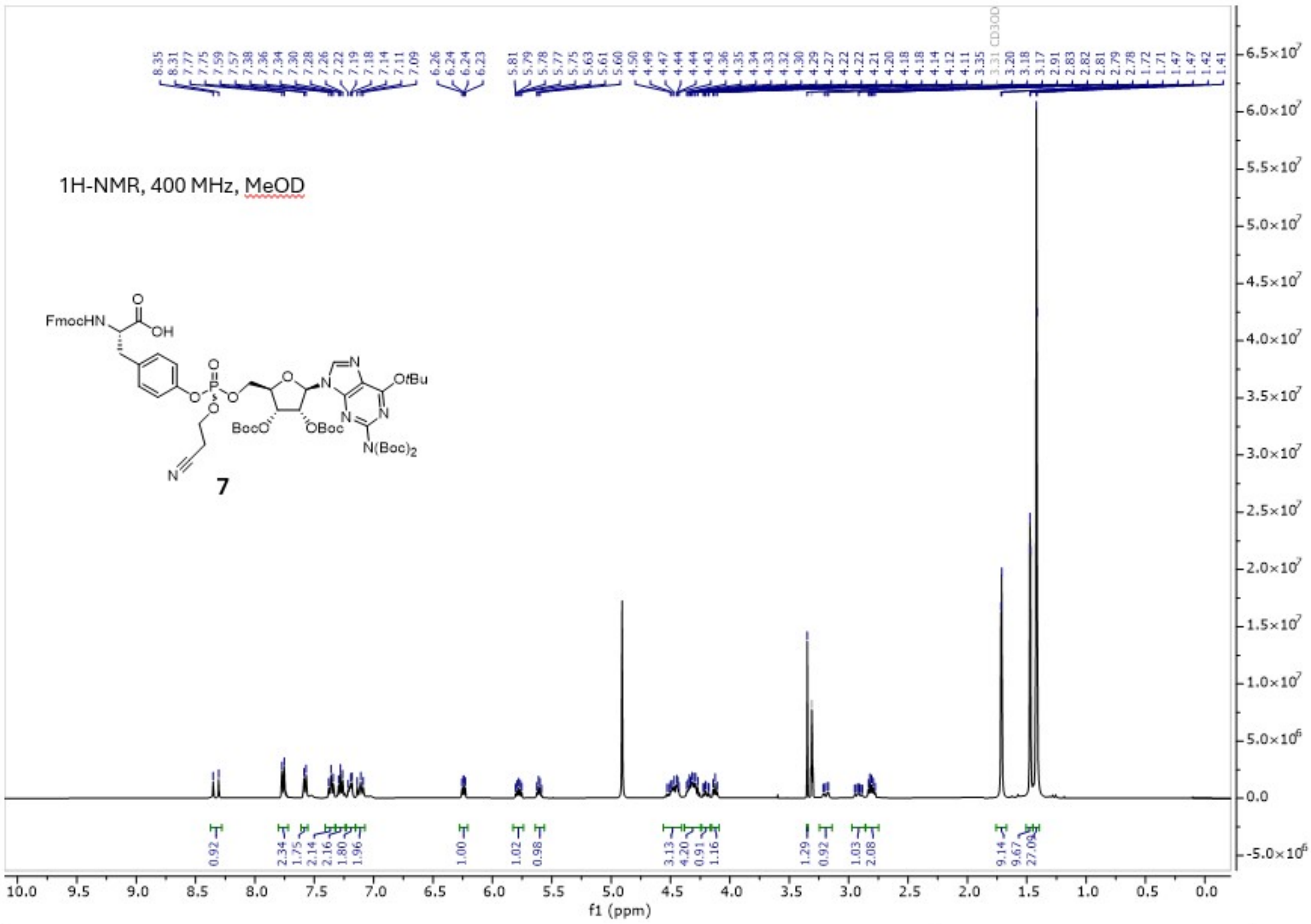
1H-NMR, 400 MHz, MeOD
7
f1 (ppm)

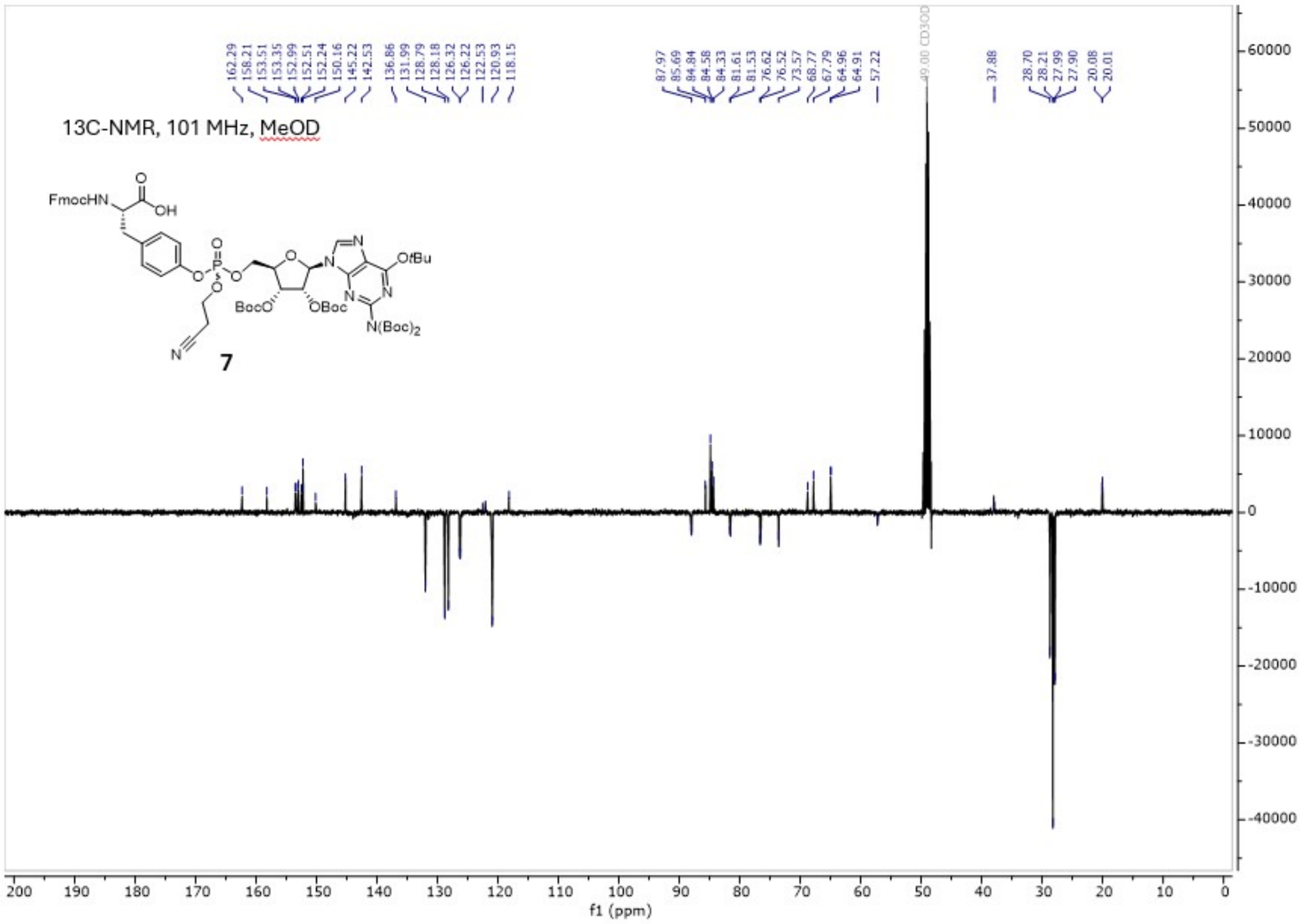
13C-NMR, 101 MHz, MeOD
7
f1 (ppm)

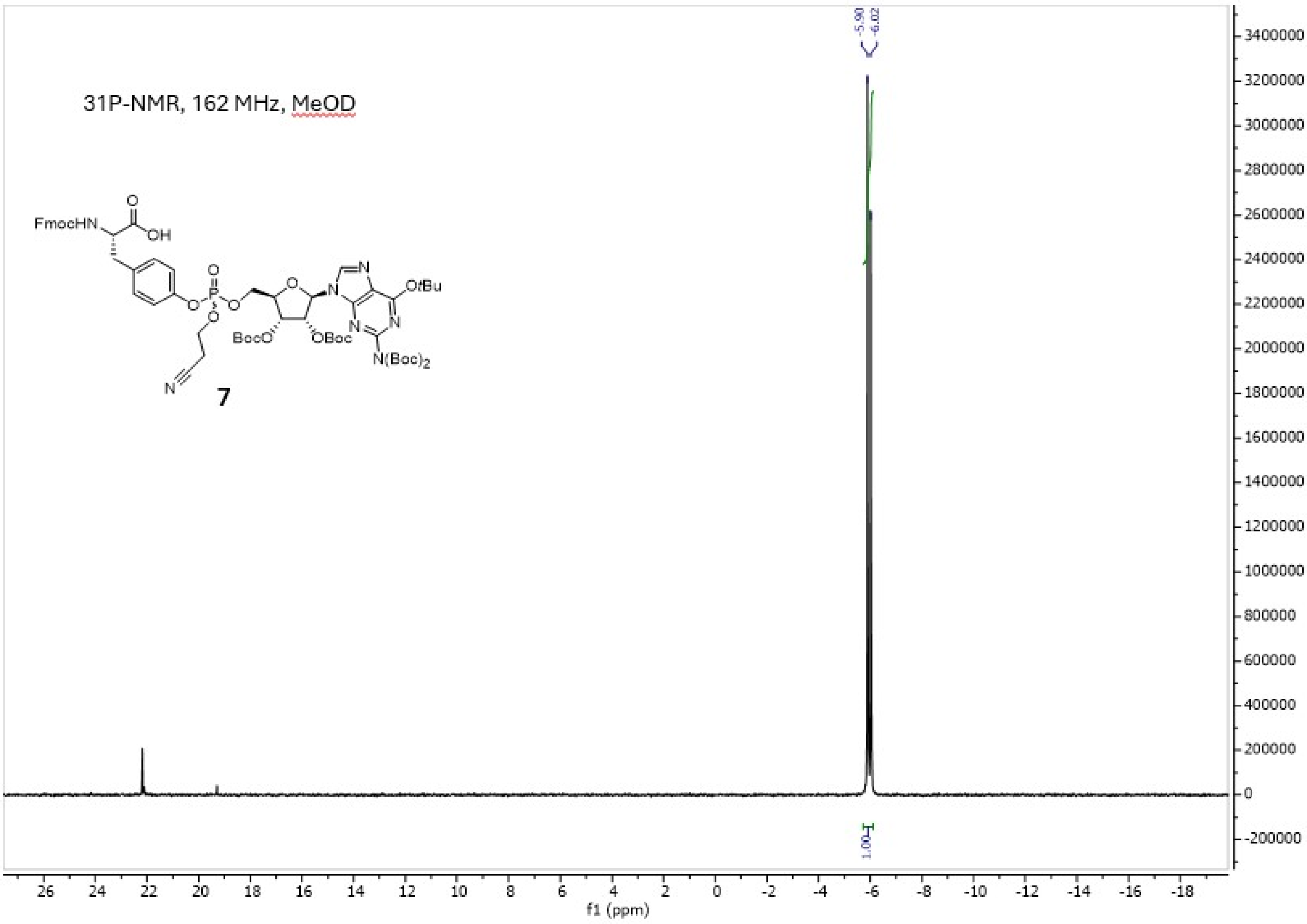

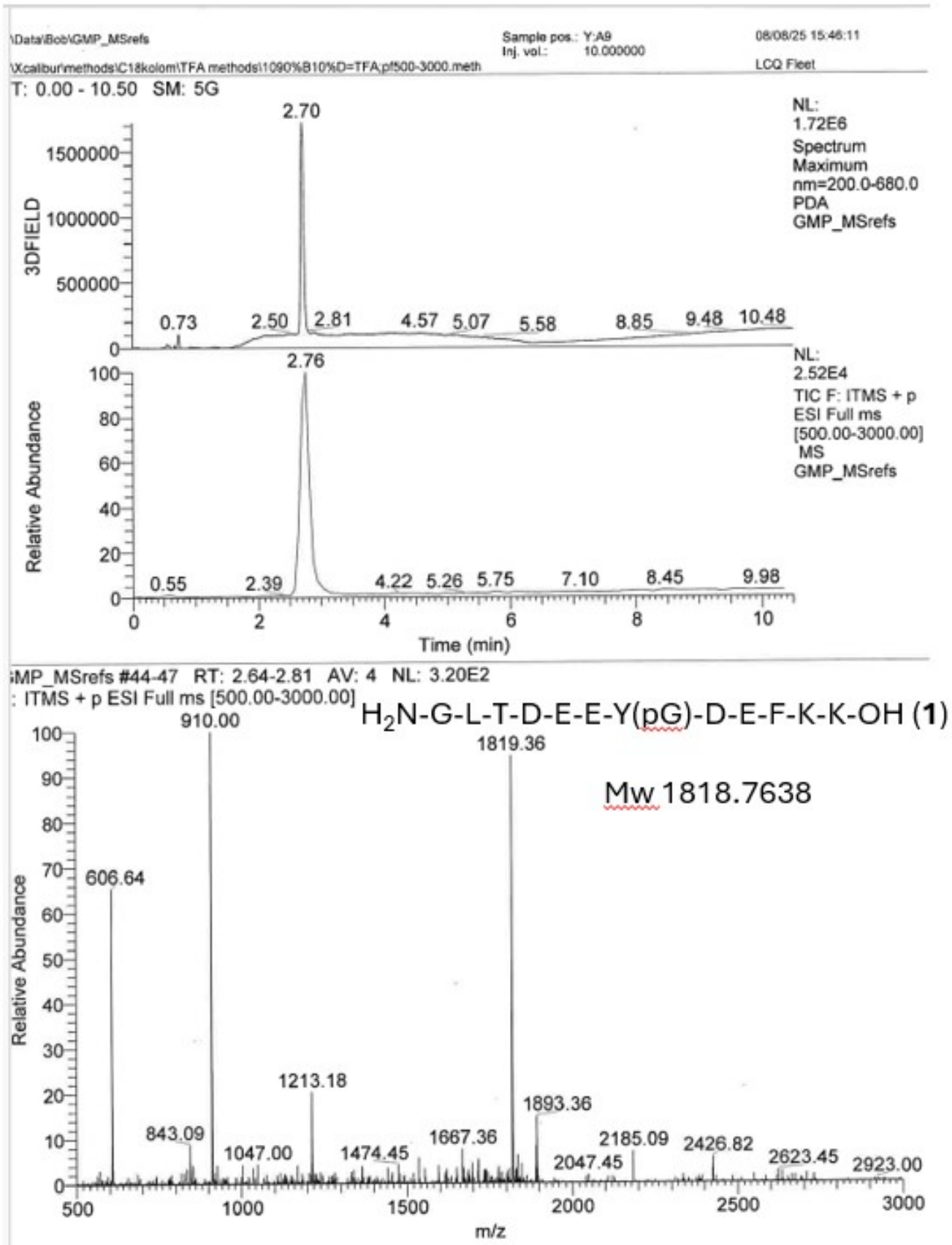

LC-MS of **1** on a C18 column from 10% MeCN in H_2O to 90% MeCN in H_2O with 0.1% of TFA. Mass range = 500 -3000.
